# Characterisation of physical and mechanical properties of seven particulate materials proposed as traction enhancers

**DOI:** 10.1038/s41597-023-02304-x

**Published:** 2023-06-22

**Authors:** Sadaf Maramizonouz, Sadegh Nadimi, William Skipper, Roger Lewis

**Affiliations:** 1 School of Engineering, Newcastle University, Newcastle upon Tyne, NE1 7RU UK; 2 Leonardo Centre for Tribology, Department of Mechanical Engineering, University of Sheffield, Sheffield, S1 3JD UK

**Keywords:** Mechanical engineering, Civil engineering, Surfaces, interfaces and thin films

## Abstract

Particulate materials are utilised in many applications to manipulate the friction between surfaces. This dataset provides the characteristics of particulates used as rail sand in the train’s wheel/rail interface (via an on-board system) to facilitate the train’s acceleration and deceleration. Seven materials are studied including Austrian rail sand, standard Great British rail sand, waste glass beads, recycled crushed glass, non-coated alumina, coated alumina, and dolomite. The main objective of this research is to provide a physical and mechanical characterisation of these granular materials in terms of their density, bulk behaviour, particle size, particle shape, hardness, reduced modulus, and mineralogical properties. In particular, three-dimensional raw and post-processed micro-computed tomography images of more than 1200 particles are shared. The results provide a detailed dataset which can be used in ongoing and future experimental and numerical investigations studying the role of particulates in the wheel/rail interface.

## Background & Summary

Particulate materials of various origins with diverse characteristics are omnipresent in natural settings and industrial applications. They are an integral part of different fields such as geology, biology, pharmaceutics, mechanical, civil, and chemical engineering as well as mining and rail industries^1^. In many cases, these particles transfer through, and interact with, the gas or liquid surrounding them and experience forces and moments highly dependent on the particles’ geometries; an example of this is the drag force acting on the particles, dictating most of their dynamics. There are numerous studies on how to model and/or estimate the drag force of non-spherical particles and the discussion is ongoing. However, one important consensus between many of them is the need for the particle properties, characteristics, geometry and shape descriptors to be already known^2^.

The particulate materials can also be used as friction modifiers. One example is the rail-sanding application in the railway industry where due to the presence of a contamination layer such as leaves, oil, or water on the surface of the rail, low adhesion between the wheel and rail occurs (In the railway industry “adhesion” or “adhesion coefficient” is defined as the amount of traction present when the wheel-rail contact enters partial slip. In this paper, the terms are used interchangeably). This can hinder train braking which poses safety risks, that in the worst case could lead to collisions. Poor acceleration also results which causes train delays. To counter the effects of low adhesion, sand particles are sprayed onto the surface of the rail ahead of the wheel. As the train moves, the wheel passes over the sand particles and crushes them and subsequently increasing the friction between the wheel and the rail^3^.

Despite the advantages of rail-sanding, there are some drawbacks. One is the low efficiency of particle entrainment^3^. Increasing the efficiency of rail-sanding calls for reliable and predictive models and strategies for estimating and measuring friction between each two components, which in turn needs detailed information on the particles’ characteristics and geometries. Another disadvantage of rail-sanding is the limited sand resources which makes it an unsustainable option in the near future. Finding sustainable alternatives for sand requires knowing the properties and characteristics of the particulate material which can be used in rail-sanding^4^.

Here, seven different particulate materials including standard British rail sand, Austrian rail sand, waste glass beads, recycled crushed glass, non-coated alumina, coated alumina, and dolomite are characterised based on their density, bulk behaviour, size, shape, mechanical, and mineralogical properties. In some cases, the data is compared to the standard British rail sand as a benchmark due to its wide usage in the British rail industry.

The data presented in this work offers a substantial database which may prove useful for any scientist and engineer working in one of the many fields dominated by particulate materials. The data included in this dataset has the potential to be utilised in both experimental studies and numerical investigations of the particulate materials and their interactions with their surroundings.

## Methods

### Preparing the samples

Each of the seven materials characterised here were obtained and prepared as follows:

- British rail sand was acquired from Network Rail, United Kingdom.
- Austrian rail sand was obtained from Virtual Vehicle, Austria.
- Waste glass beads were acquired from Potters-Ballotini Co. Ltd., Japan.
- Recycled crushed glass was produced by washing and crushing various types of glass bottles. The particles were sieved using three different mesh sizes of 2, 1.18 mm, and 600 µm mesh sieve.
- Non-coated alumina and coated alumina were acquired from L.B. Foster Rail Technologies, United Kingdom.
- Dolomite was acquired from a quarry in the Northeast of England and was sieved using three different mesh sizes of 2 mm, 1.18 mm, and 600 µm.

### Density

Particles’ density was assessed using the gas jar method following BS1377-2:1990^5^. In this method, two glass cylinders, two ~400-gram samples of each material, distilled water and an end-over-end shaker were utilised. Four mass values were measured during this experiment. *m*_1_ is the mass of each empty glass cylinder with its glass cap on, *m*_2_ is the mass of each glass cylinder containing the sample, *m*_3_ is the mass of each glass cylinder containing the sample and filled to the brim with distilled water after being tumbled for 20 minutes by the end-over-end shaker, and *m*_4_ is the mass of each glass cylinder filled to the brim with distilled water. The density of each specimen can be calculated using the following formula:

1 $$\frac{{m}_{2}-{m}_{1}}{\left({m}_{4}-{m}_{1}\right)-\left({m}_{3}-{m}_{2}\right)}$$

It is worth mentioning that the available amount of two candidates, namely, Austrian rail sand, and waste glass beads, were not sufficient for performing the gas jar method.

### Bulk characteristics

The particles’ Angle of Repose (AoR), the angle a pile of granular material produces relative to the horizontal plane, is estimated as a measure to characterise their bulk behaviour. AoR can be used to quantify the “flowability” of granular materials resulting from the particles’ size and shape, along with the interparticle friction and adhesion. There are various methods of measuring AoR, however, none of them are defined as the standard method.

Here, in order to estimate the candidates’ AoR, the procedure proposed by the technical committee of the International Society for Soil Mechanics and Geotechnical Engineering (ISSMGE TC105) as a part of a round robin test was utilised^6^. For each material, three experiments were conducted, and photos were taken using a digital camera (SLR camera Canon (Tokyo, Japan) EOS 60D 18 MP CMOS) with EF-S 18–200-mm lens. Image analysis for measuring the angles were done using the open-source software Fiji-ImageJ^7^.

It is worth mentioning that the available amount of two candidates, namely, Austrian rail sand, and waste glass beads, were not sufficient for measuring their AoR.

### Size and shape

The sizes and shapes of particles for each material were characterised by sieving and X-ray micro-Computed Tomography (µCT) scans, respectively. The μCT system (SkyScan 1176) is located at the Preclinical *in-Vivo* Imaging Facility at Newcastle University Medical School, United Kingdom. The scans were performed with a source current of 357 μA and a voltage of 70 kV.

In order to create the 3D geometry of the particles, the μCT images were reconstructed to produce greyscale cross-sectional slices with a voxel side length/image resolution of 8.81 μm resulting in the 3D images to have approx. 7444 × 7444 × 7117 voxels.

To increase the computational efficiency of image processing, the μCT images were resized with a factor to reduce the size of the 3D matrix while conserving the particles’ size and shape characteristics^8^. The exact value of scaling factor and resulting voxel size length/image resolution are noted in the dataset.

After analysing the μCT image, the particles’ 3D geometries are created which are included in this dataset as *.STL files. The shapes and geometries of the particles from each material are classified using Zingg^9^ Plots and the particles’ surface area and volume as well as the particle shape descriptors including elongation, flatness, sphericity, and convexity were evaluated utilising the SHAPE code by Angelidakis *et al*.^1^. All these data are also included in this dataset.

### Mechanical properties

To measure the mechanical properties of the candidate materials, nano-indentation tests were performed. Samples of all seven materials were mounted on a steel stub and a nano-indentation instrument (NanoTest Vantage) with a diamond Berkovich indenter was used for the experiments. The NanoTest Vantage is fully compliant to all relevant international nanoindentation standards including ISO14577 (the ISO standard for instrumented indentation) and ASTM E2546–07. The ISO14577 standard specifies the methods for calibration of the test instrument and verification using reference samples and the NanoTest Vantage is regularly calibrated to ensure compliance with the standard. The indenter used in the measurement was a commercial Berkovich indenter which is a three-faced pyramidal indenter most commonly used for nanoindentation. The tip of the Berkovich indenter is not infinitely sharp, but instead is rounded, with a tip radius of 160 nm. A fused silica reference sample was used to calibrate the tip shape of the indenter and determine the area function (i.e., tip sharpness) prior to testing.

For each test, the maximum load, loading time, unloading time, and maximum load hold were set to 80 mN, 8 s, 4 s, and 10 s, respectively. The NanoTest applies the loading force in the horizontal direction. The sample is affixed to a sample holder and can be moved in all six directions using the three-axis stage movement system on the NanoTest. Each of these stages has an optical encoder built-in to precisely measure the movement of the stage with 20 nm resolution. The repositioning accuracy is <0.5 µm. The indentation grids were set using the integrated optical microscope in order to avoid certain areas of the sample, e.g., large cracks.

The high surface roughness of some samples (especially with smaller particle sizes) results in a high degree of variability for the hardness and reduced modulus values which is shown in the large standard deviation of the data. To eliminate the unfavourable effects of the surface roughness, for each of the seven materials the experiments were repeated to obtain at least 10 reasonable indentations. In this context, ‘Reasonable’ refers to the shape of the depth vs load hysteresis curve. For samples with high surface roughness, the position where the indenter contacts the sample has a big influence on the measured properties. Ideally (for a perfectly flat sample) the indenter always contacts the sample at 90 degrees and all the indentation curves are closely grouped. For samples with a very high roughness, there is a lot more scatter. For some indentations, the contact can be so ‘off-normal’ that the indentation curve looks different to the rest. This often seen as a very large, anomalous increase in depth at the beginning of indentation or just an unconventional indentation curve which yields hardness and modulus results very different to the average for that experiment.

Materials’ hardness and reduced modulus were the results of the nano-indention tests which are defined as the ratio of maximum load to indent area, and the slope of unloading, respectively.

### Mineralogical properties

To investigate the mineralogical characteristics of the candidate materials, phase identification of the particles was performed using powder X-ray diffraction (XRD). For XRD experiments, a small sample of each of the seven materials was ground to a fine powder and placed inside the sample holder of the diffractometer (Bruker D2 Phaser with LynxEye detector using Cu Kα radiation). Before the main measurements were carried out, a preliminary scan (with 2θ between 5–100°) was run to check for low angle peaks.

The diffractometer parameters including divergence slit, 2θ range, step size, and step-1 were set to 1.0 mm, 10–100°, 0.033°, and 0.5 s, respectively. An Ni filter was used to reduce Kβ radiation.

The peaks for each material are compared to reference patterns for compounds/materials present within the Crystallography Open Database (COD). The parameters a (Å), b (Å), and c (Å) which are lengths of the vectors that lie along the edges of the parallelepiped enclosing a unit crystal cell, and the parameters α (°), β (°), and γ (°) which represent the angles between the vectors b and c, vectors a and c, and vectors a and b, respectively, are all provided in the dataset.

It is worth noting that waste glass beads were shown to be amorphous thus no phases could be identified.

## Data Records

All the data records are publicly available at 10.25405/data.ncl.21710669^10^.

The dataset is comprised of one spreadsheet and one folder. The spreadsheet contains five individual sheets, one for each of the investigated properties including density, bulk behaviour (AoR), particle size distribution, mechanical properties (Nano-Indentation), and mineralogical properties (XRD). The folder is dedicated to the shape characterisation and is comprised of one *.PDF file which includes the graphs for the shape characterisation, ten items including British and Austrian rail sand, dolomite, waste glass beads, coated (coarse and fine) and non-coated alumina, and recycled crushed glass (three sizes). Each of these items contain more than 50 MATLAB data files corresponding to a single particle of the aforementioned material which has been scanned through µCT and includes the three-dimensional geometry of the particle along with all the particle data and characteristics. These data files are saved as “particle types” and can be loaded and read in the MATLAB interface. If access to MATLAB is not available, the free and open-source alternative, GNU Octave (https://octave.org/), can be used to access the data. The three-dimensional geometries of each single particles are also included as *.STL files in the folder corresponding to each candidate material.

**Fig. 1:**
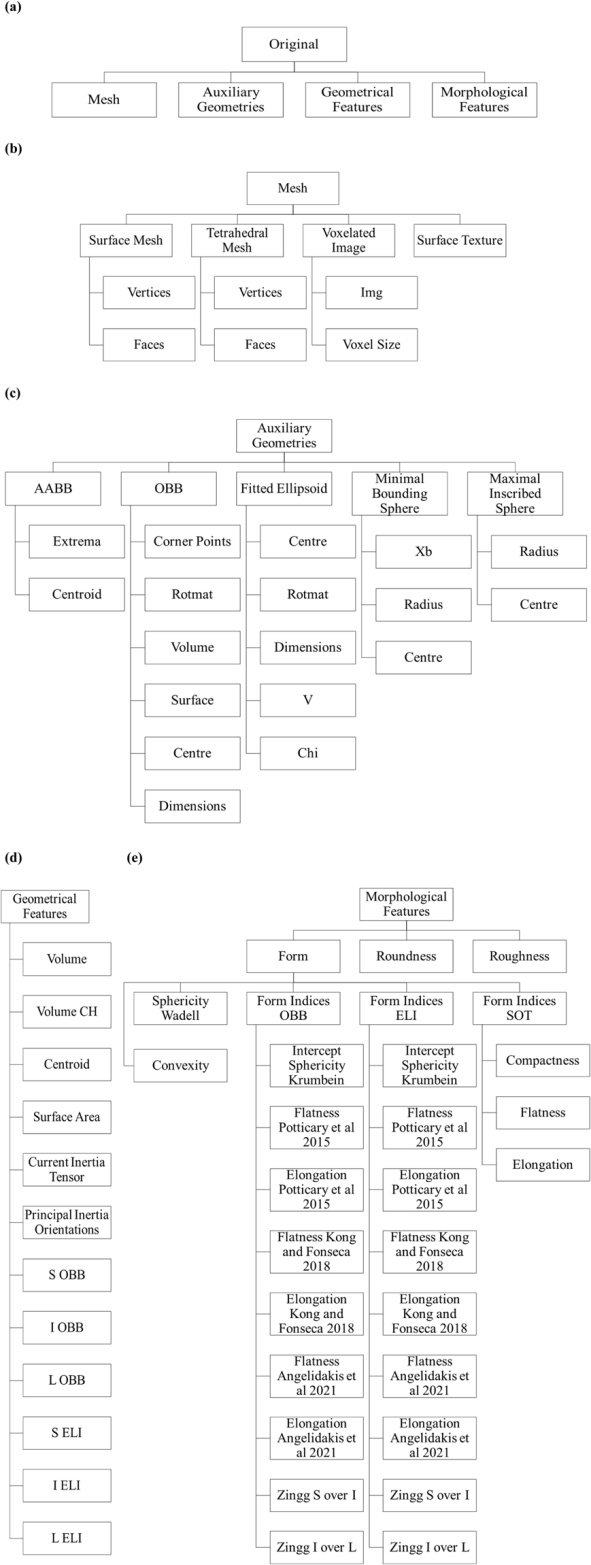
Data structure of the particle MATLAB data files for (**a**) overall view, (**b**) mesh, (**c**) auxiliary geometries, (**d**) geometrical features, and (**e**) morphological features.

Figure 1 shows the structure of the data included in the MATLAB data files for each particle. The parameters S, I, and L are the short, intermediate, and long dimensions of the particle geometry. It is worth noting that the indicators AABB, OBB, ELI, and SOT stand for axis-aligned bounding box, oriented bounding box, fitted ellipsoid, and surface orientation tensor, respectively. Additionally, ‘*Img*’ represents the three-dimensional matrix containing the µCT images of the particle, ‘*Rotmat*’ is a 3 × 3 matrix, where the columns correspond to the vectors pointing along the short, intermediate and long axes, respectively, ‘*V*’ contains 10 parameters that can be used to define an ellipsoid in its algebraic form, and ‘*Chi*’ is the root mean square error of the distance between the particle vertices and the fitted ellipsoid.

In order to generate the three-dimensional geometry of each particle, the MATLAB function “savestl (node, elem, fname)” can be utilised which is provided by the Iso2Mesh code^11^ and needs three inputs: particle vertices as node, particle faces as elem, and file name as fname and will save the particle geometry as an *.STL file which can be used in any computer-aided design (CAD) software and numerical simulation code.

## Technical Validation

For all tests, two main points have been considered. Firstly, that the samples of each candidate material are produced in a manner to represent the whole batch. Secondly, that each test is repeated enough times to obtain reasonable data.

To measure the density of the materials the British Standard (BS1377-2:1990^5^) was followed step by step and as accurately as possible.

The samples for particle shape characterisation through μCT imaging were prepared with utmost care to ensure two factors. One, that there are enough number of particles for each material candidate to warrant adequate data. Two, that there are no touching particles to achieve accurate image analysis. For this purpose, a long piece of tape was fixed to the work surface, the particle sample was lightly sprinkled on top, and the connecting particles were disconnected using a fine tip tweezer. Then, another long piece of tape was placed on top of the sprinkled sample with the sticky side down sandwiching the particles. This ensured that the particles will not move during testing. Finally, the prepared sample was rolled to form a cylinder and placed in an individual cylindrical container for imaging.

For nano-indentation tests, the samples were chosen with utmost care to provide particles suitable for nano-indentation testing. For this purpose, a large number of particles were spread over a white surface which was used for all the candidate materials except for waste glass beads for which a black surface was utilised. The particles were then thoroughly examined using a magnifying glass to detect the ones with at least one flat surface. For the candidate materials with smaller sizes, at least 15 particles and for candidate materials with larger sizes, three large particles are chosen, all with flat surfaces. These chosen particles were plucked by a tweezer and mounted on the cylindrical steel stub using CYL Cyanolube adhesive. The particles were mounted with their flat surface on top and positioned horizontally to enable accurate indentations. It is worth noting that in order to obtain more reliable values of hardness and reduced modulus, the samples can be mounted and then polished flat to significantly reduce the surface roughness. These values, however, will not be representative for the purpose of our research.

The results of the XRD tests were compared to the data provided by Scanning Electron Microscopy (SEM) with Energy Dispersive X-Ray Analysis (EDX) (located at School of Engineering, Newcastle University, United Kingdom) to ensure their accuracy.

## Usage Notes

For each material candidate more than 50 MATLAB data files are presented. Each file corresponds to one particle which has been scanned through µCT and includes the three-dimensional geometry of the particle along with all the particle data and characteristics. Particles’ geometry and characteristic data can be extracted using the SHAPE code by Angelidakis *et al*.^1^.

## Supplementary information

Supplementary Information

## Data Availability

Shape characterisation of particles and calculation of shape descriptor parameters including surface area, volume, elongation, flatness, sphericity, and convexity are performed utilising the SHAPE code by Angelidakis *et al*.^1^ publicly available from the link below: https://github.com/vsangelidakis/SHAPE. Image analysis is performed using the open-source software Fiji-ImageJ (1.53c)^7^ and MATLAB (R2021a).
