# Supplementary material for: Characterisation of physical and mechanical properties of seven particulate materials proposed as traction enhancers
Source: Sci Data. 2023 Jun 22;10:400. doi: 10.1038/s41597-023-02304-x (PMC10287628; doi:10.1038/s41597-023-02304-x)
Supplement: Supplementary file 1 — Supplementary Information [file 41597_2023_2304_MOESM1_ESM.docx]

***Supplementary Information***

*For*

*Characterisation of Shape and Shape Descriptors of the Particulate Materials Proposed as Traction Enhancers*

Sadaf Maramizonouz^1^, Sadegh Nadimi^1^*, William Skipper^2^, Roger Lewis^2^

1 School of Engineering, Newcastle University, Newcastle upon Tyne, NE1 7RU, UK

2 Leonardo Centre for Tribology, Department of Mechanical Engineering, University of Sheffield, Sheffield, S1 3JD, UK

Corresponding author(s): Sadegh Nadimi (sadegh.nadimi-shahraki@newcastle.ac.uk)

**Table of Contents**

[Particle shape distributions 2](#_Toc135163276)

[Particle shape characterisation 3](#_Toc135163277)

[References 8](#_Toc135163278)

# Particle shape distributions

The shapes and geometries of the particles from each candidate material are classified using Zingg Plots^1^ presented below:

|  | 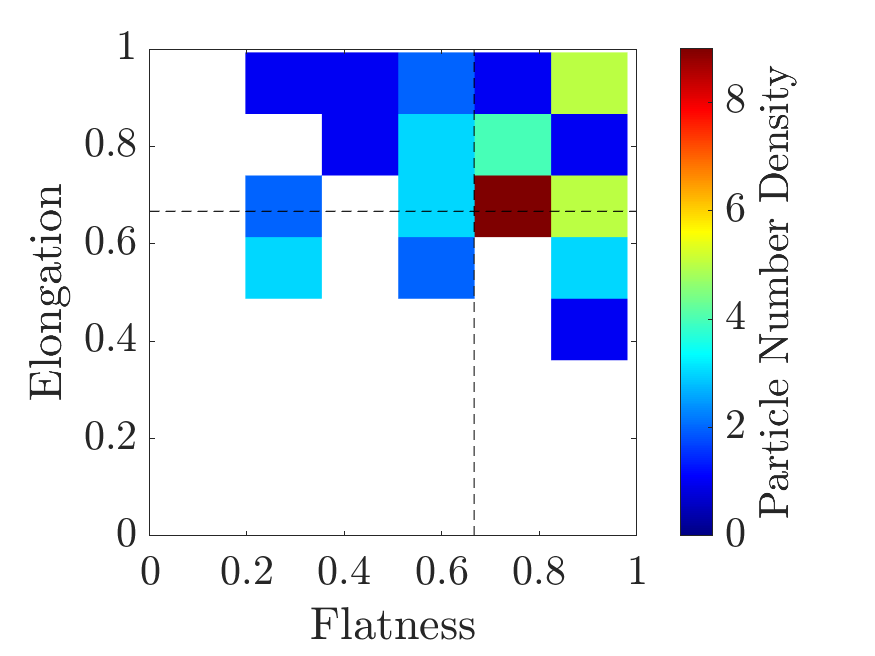**(a)** |  |
| --- | --- | --- |
| 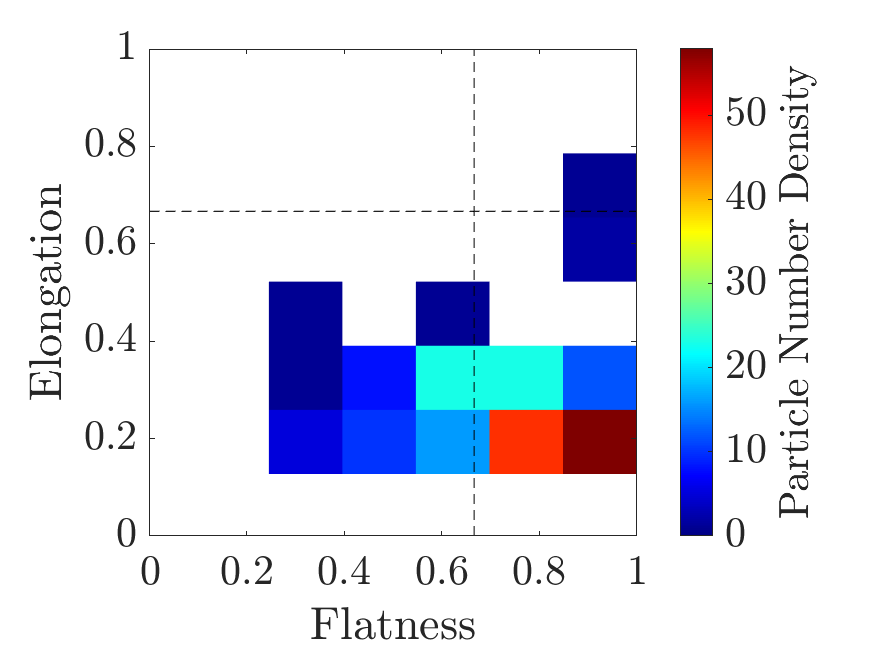**(b)** | **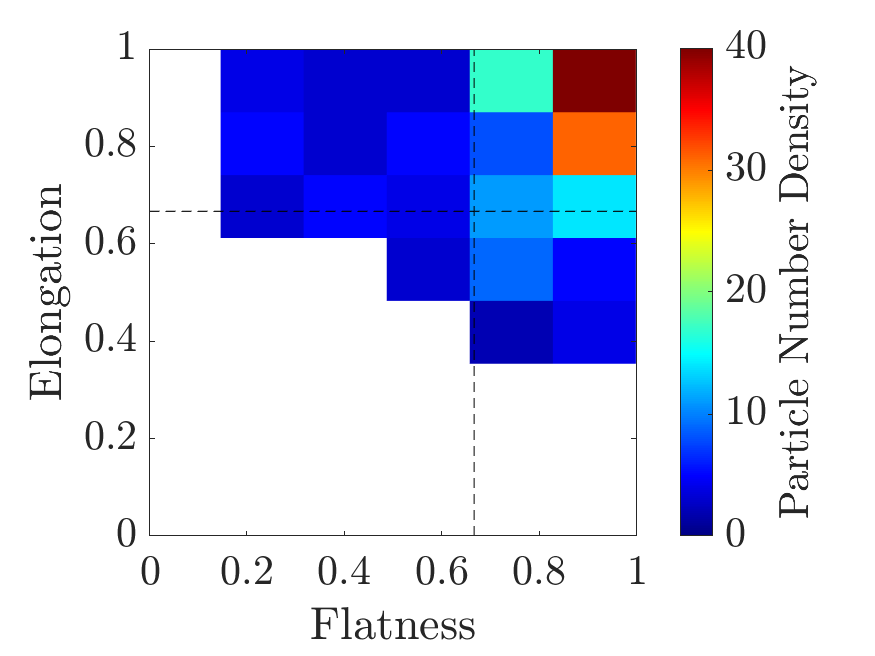** **(c)** | **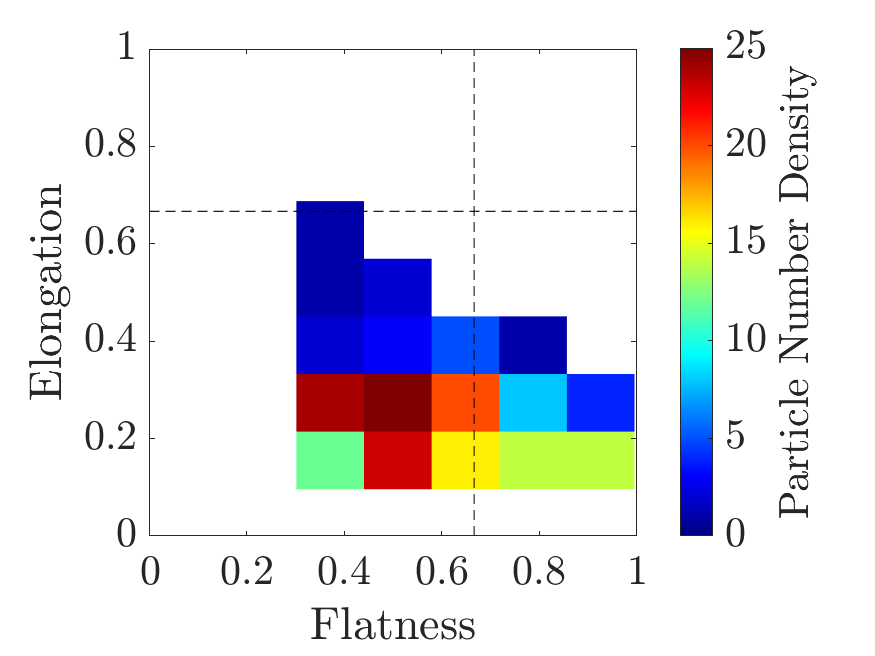(d)** |
| 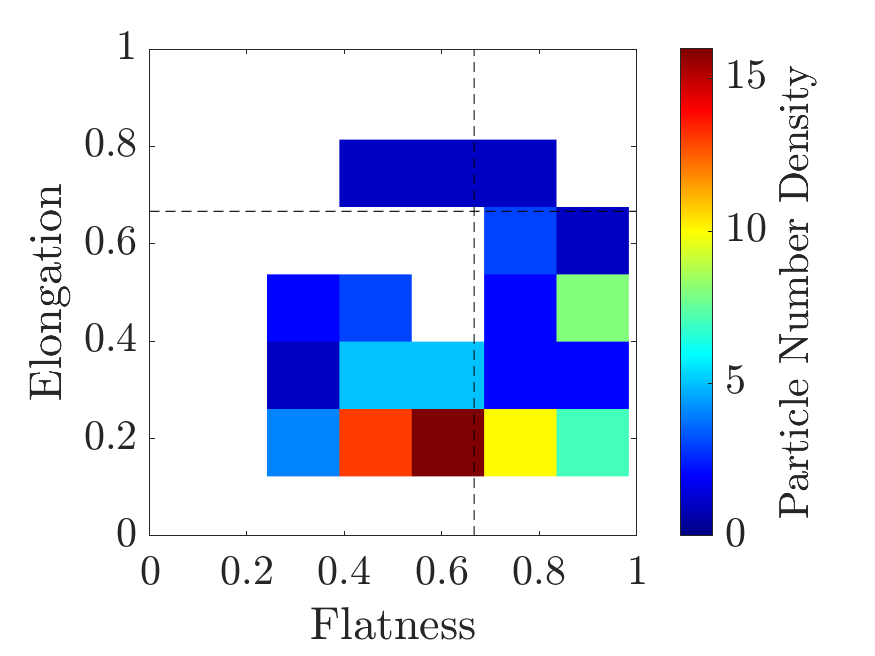**(e)** | 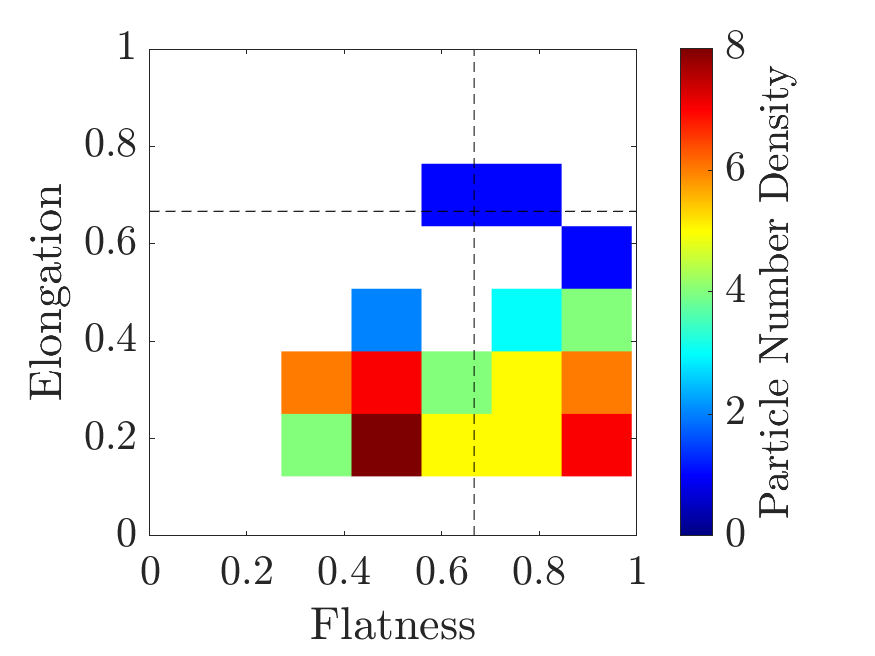**(f)** | 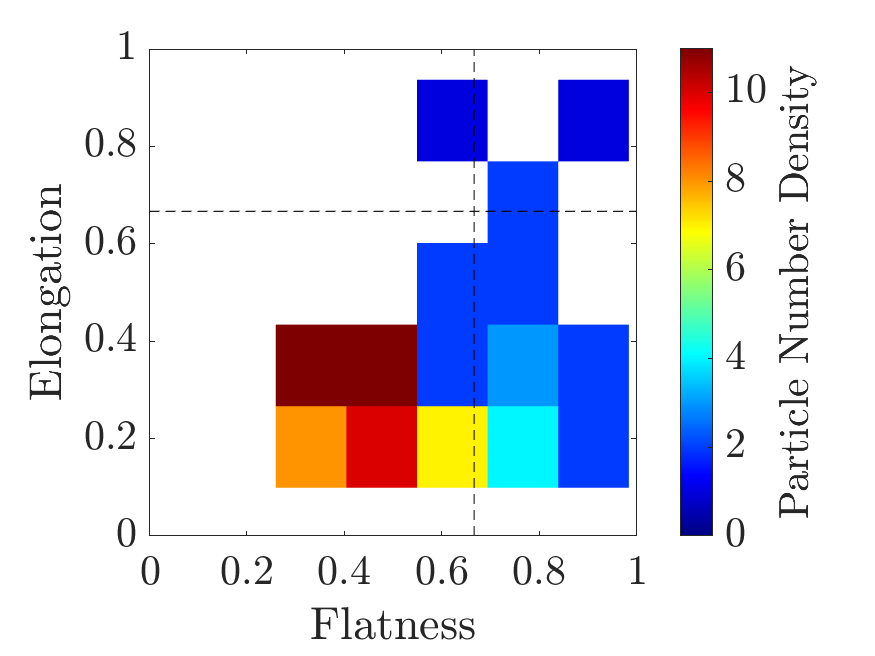**(g)** |
| 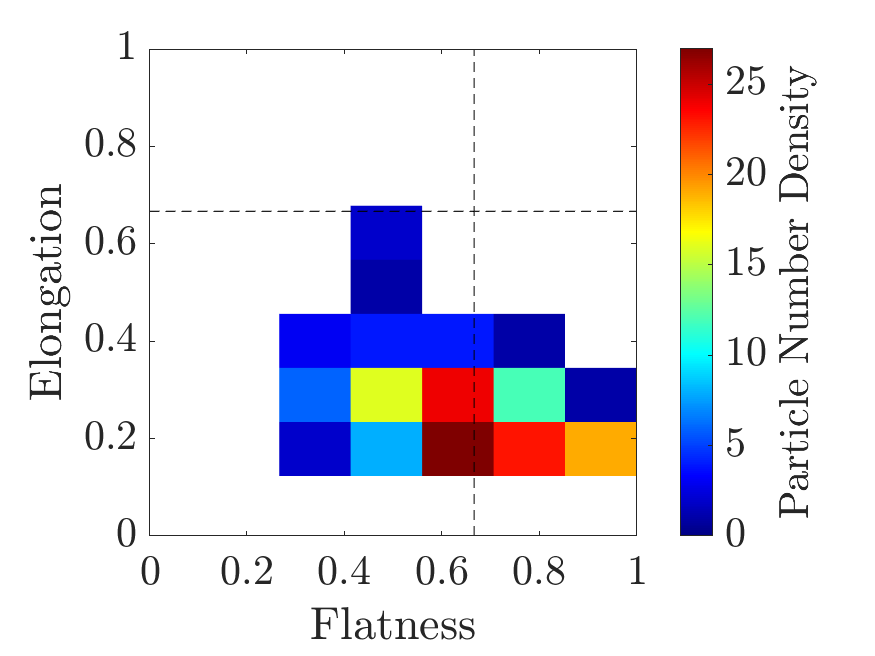**(h)** | 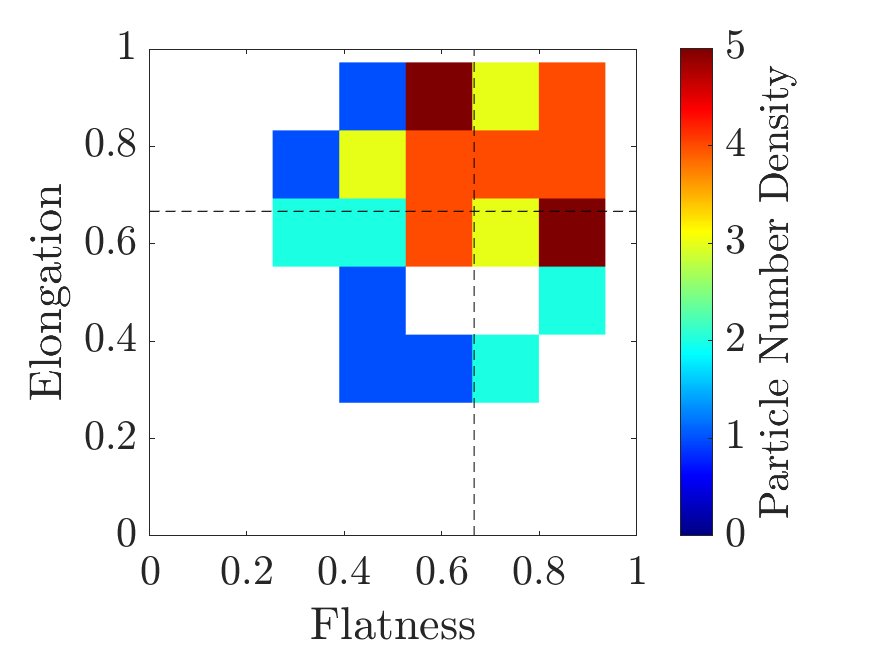**(i)** | 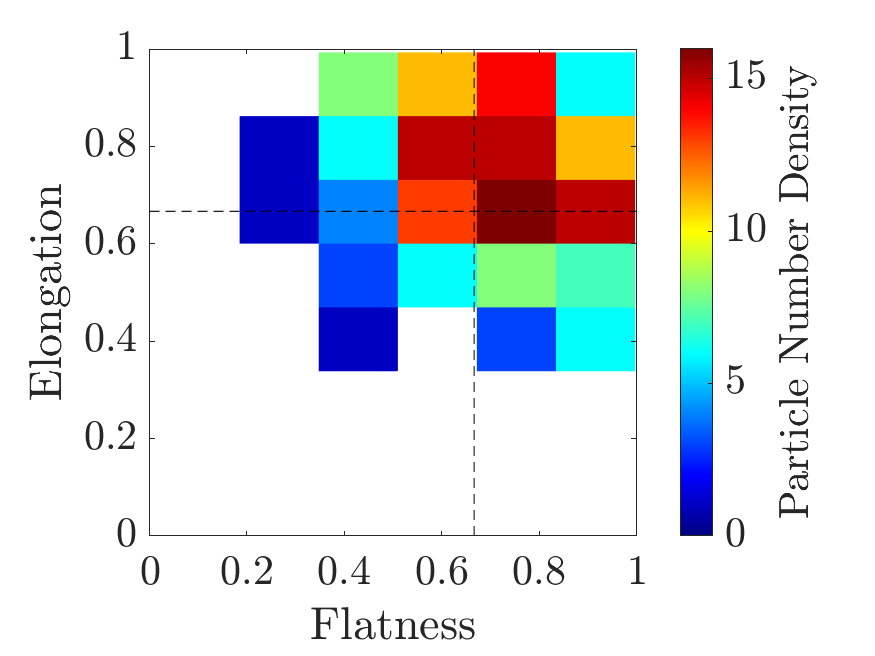**(j)** |
| *Particle shape distributions of* ***(a)****British rail sand,* ***(b)****Austrian rail sand,* ***(c)****waste glass beads,* ***(d)****dolomite,* ***(e)****Recycled crushed glass retained on 2 mm mesh sieve,* ***(f)****Recycled crushed glass retained on 1.18 mm mesh sieve,* ***(g)****Recycled crushed glass retained on 600 µm mesh sieve,* ***(h)****non-coated alumina,* ***(i)****coarse coated alumina, and* ***(j)****fine coated alumina based on flatness and elongation plotted on Zingg charts and obtained from X-ray Computed Tomography.* | | |

# Particle shape characterisation

The particles’ surface area and volume as well as the particle shape descriptors including elongation, flatness, sphericity, and convexity were evaluated utilising the SHAPE code by Angelidakis et al.^2^ and the results are shown below:

(Readers are referred to the study by Angelidakis et al.^2^ and Angelidakis et al.^3^ for more information on particle shape descriptors and classification, respectively.)

| 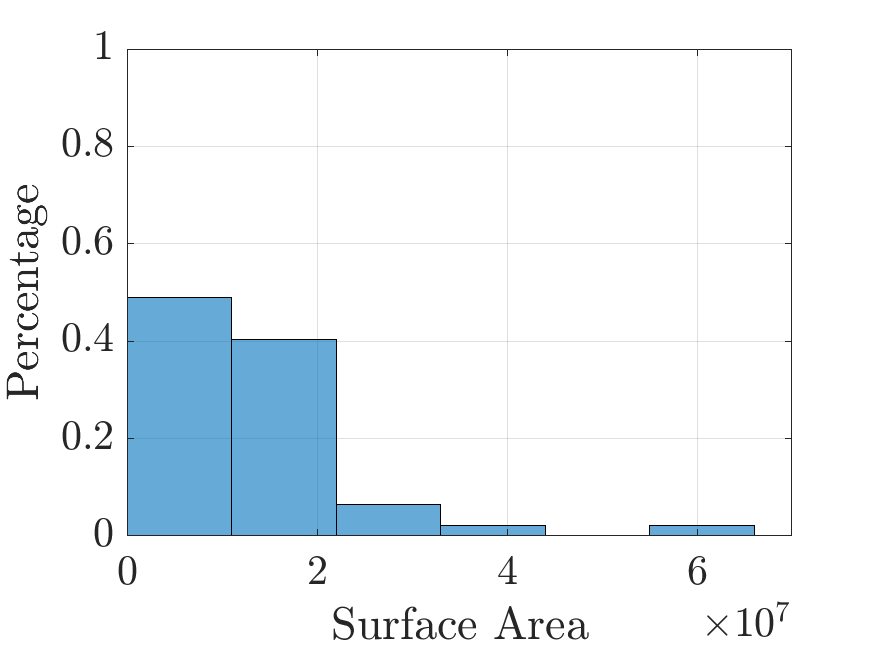**(a-1)** | | 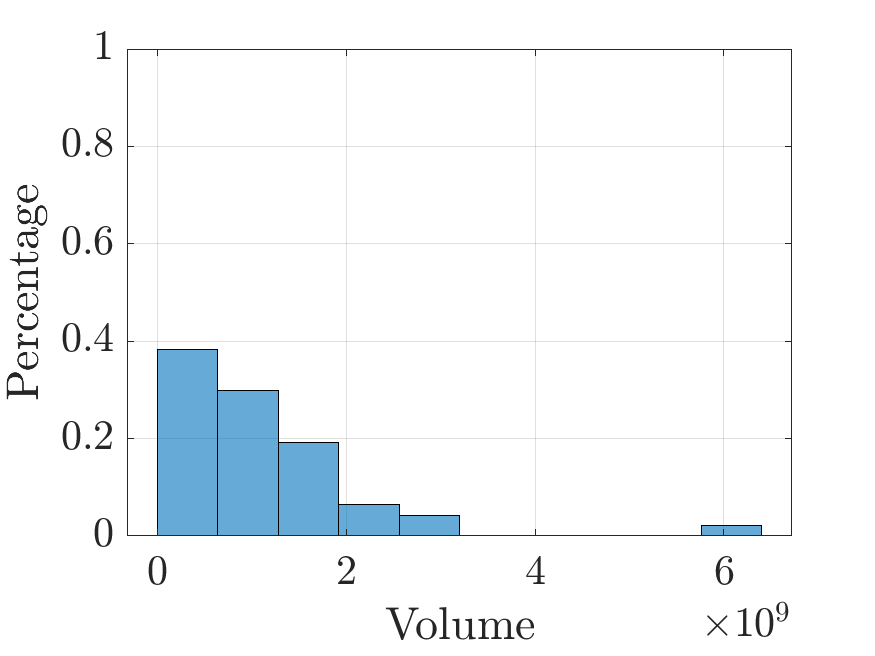**(a-2)** | |
| --- | --- | --- | --- |
| 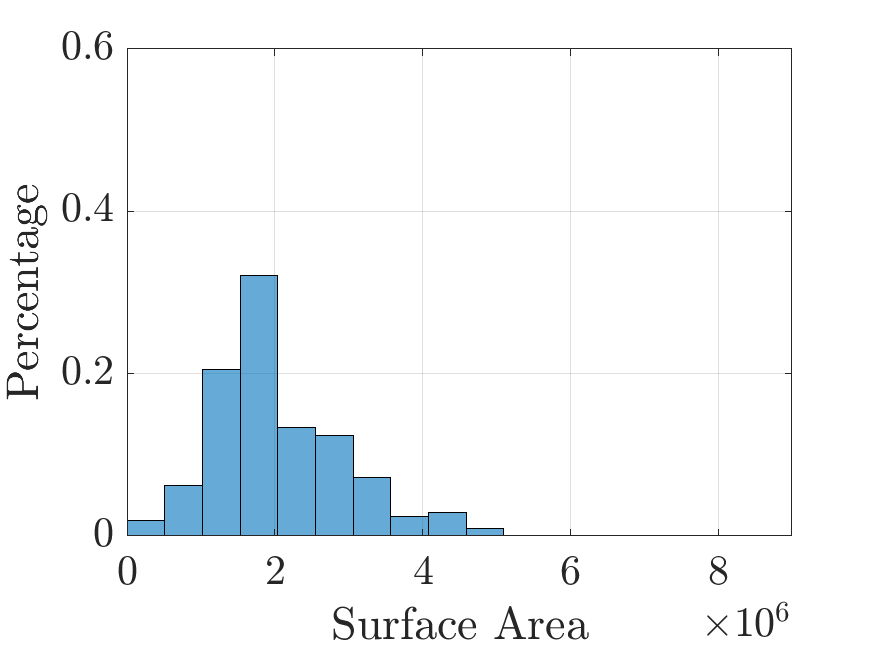**(b-1)** | | 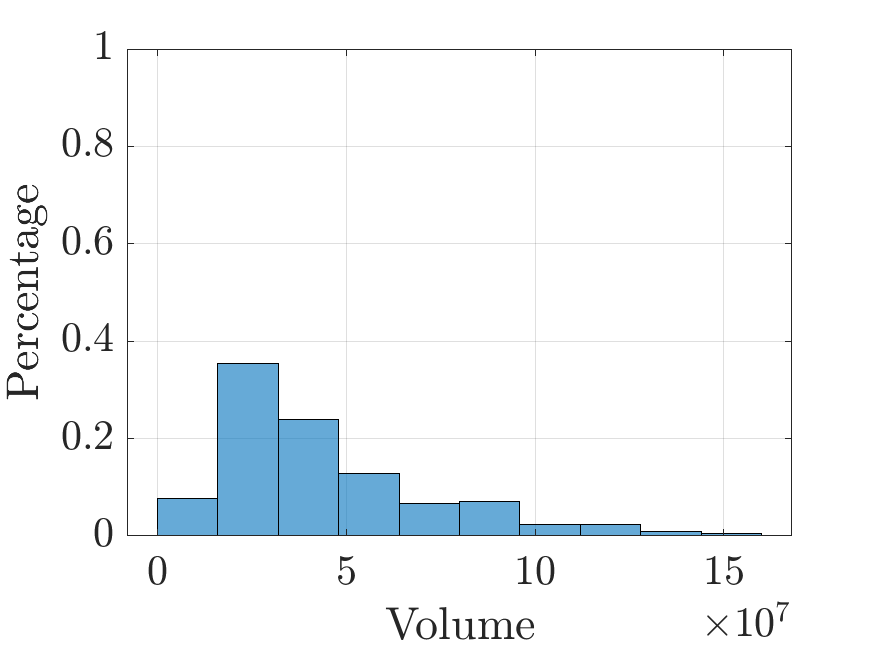**(b-2)** | |
| 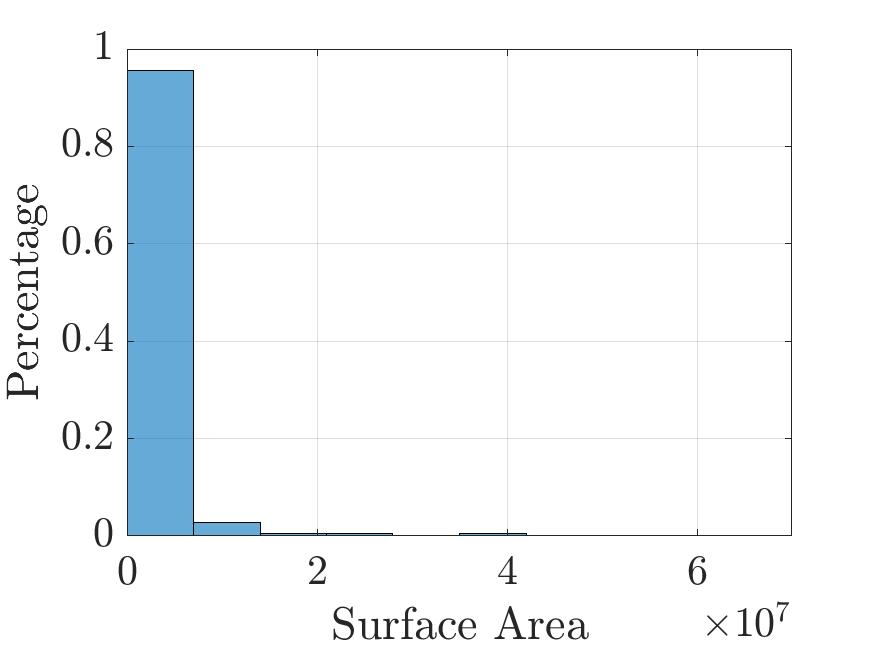**(c-1)** | | **(c-2)**  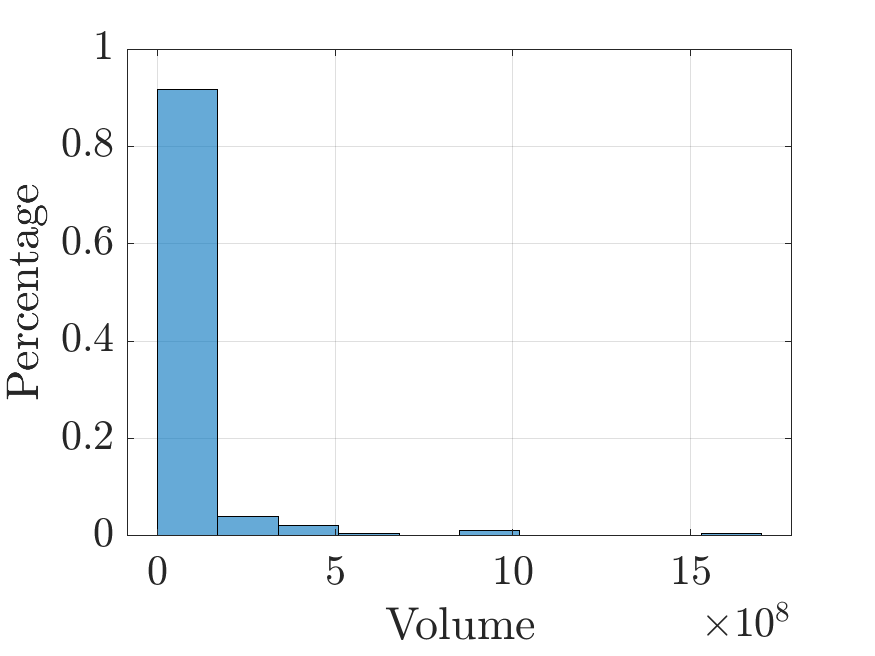 | |
| 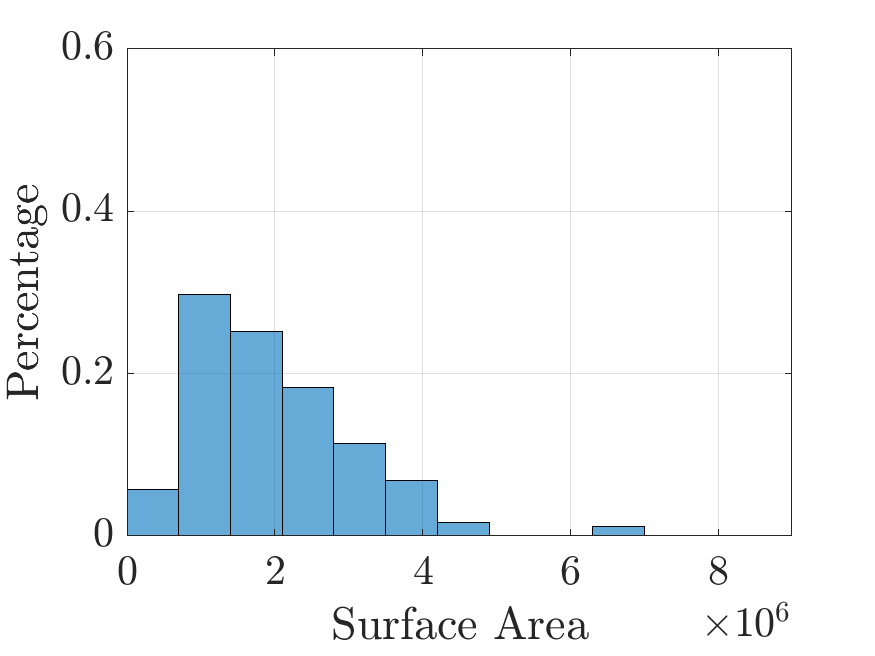**(d-1)** | | 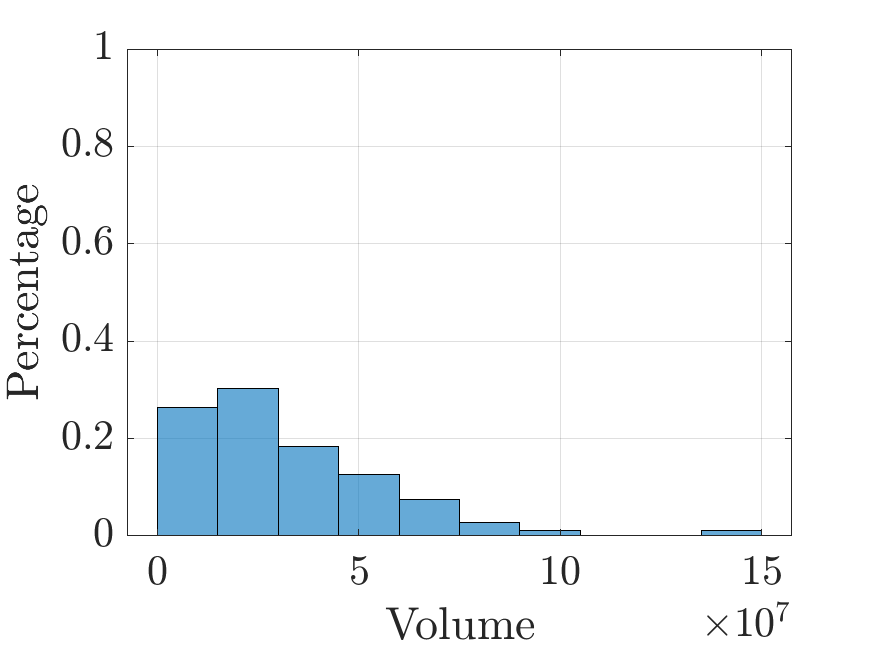**(d-2)** | |
| 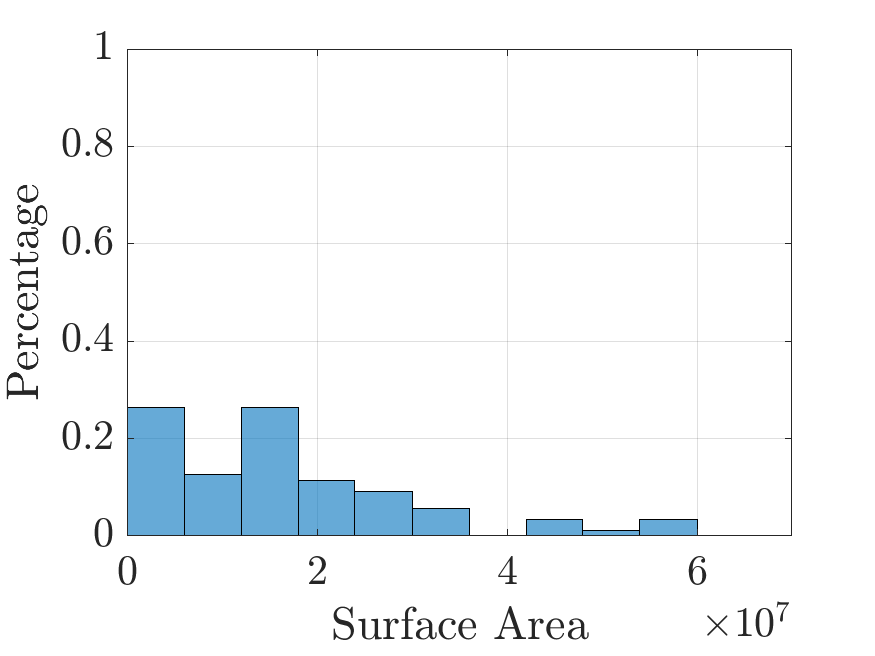**(e-1)** | | 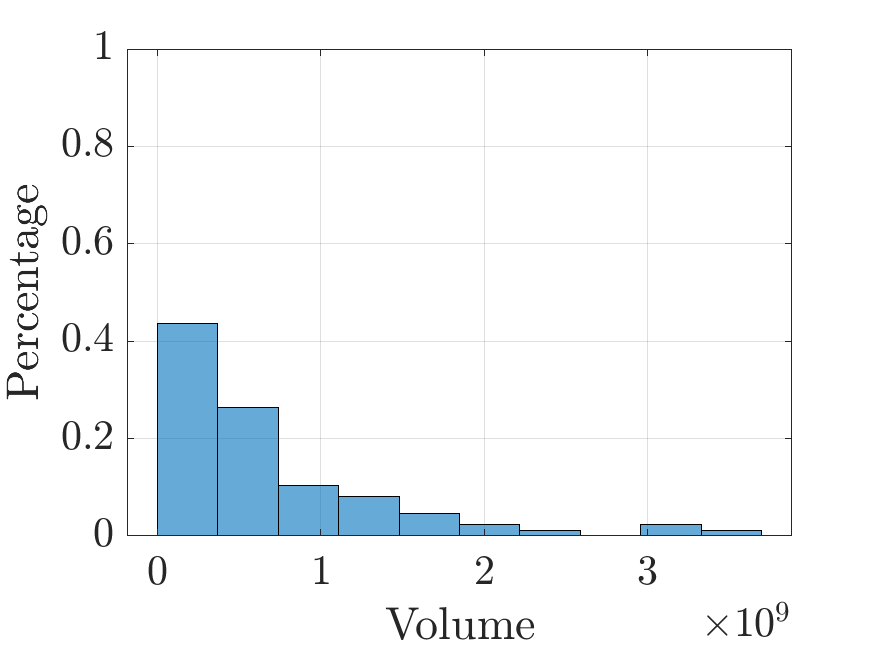**(e-2)** | |
| 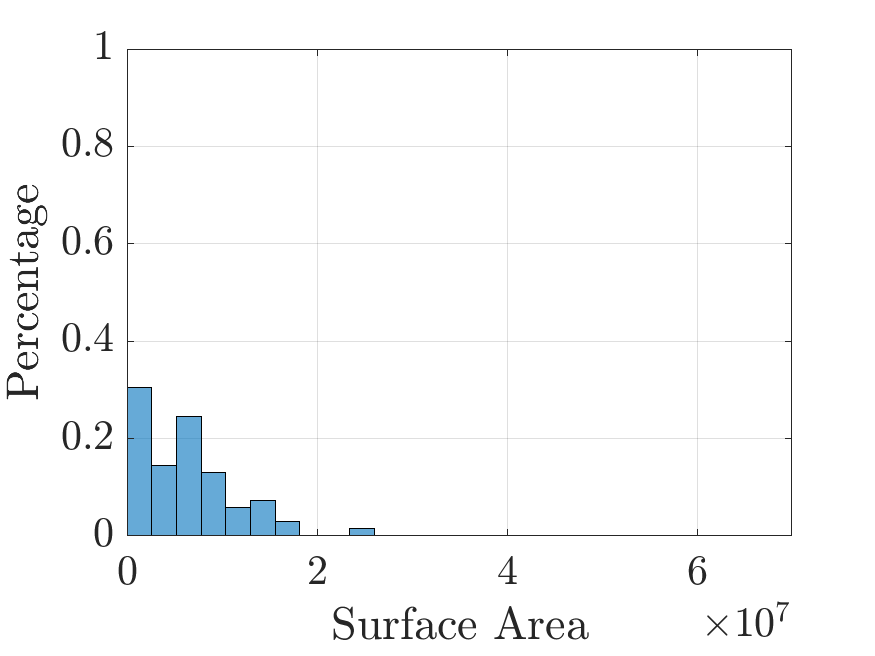**(f-1)** | | 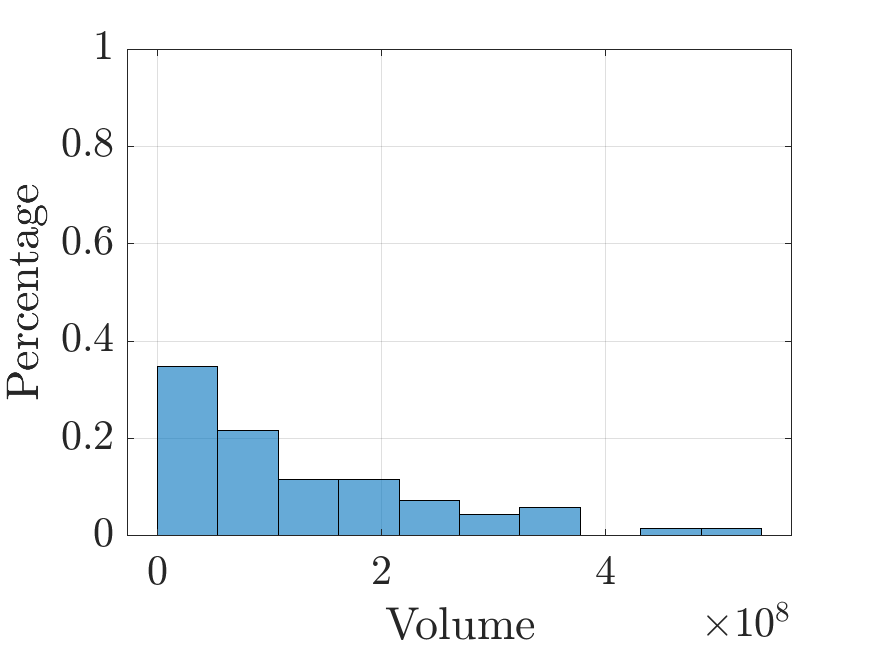**(f-2)** | |
| 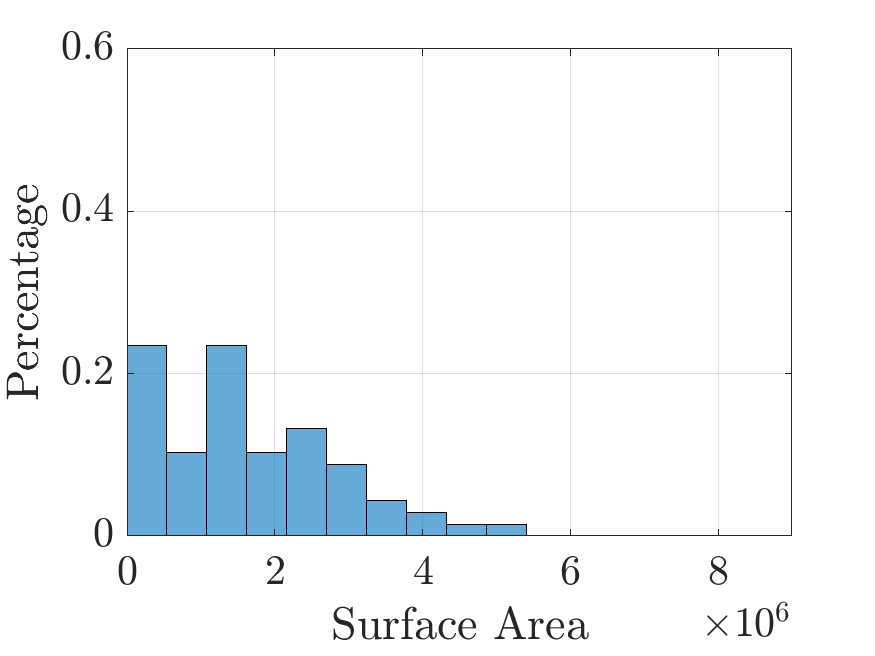**(g-1)** | | 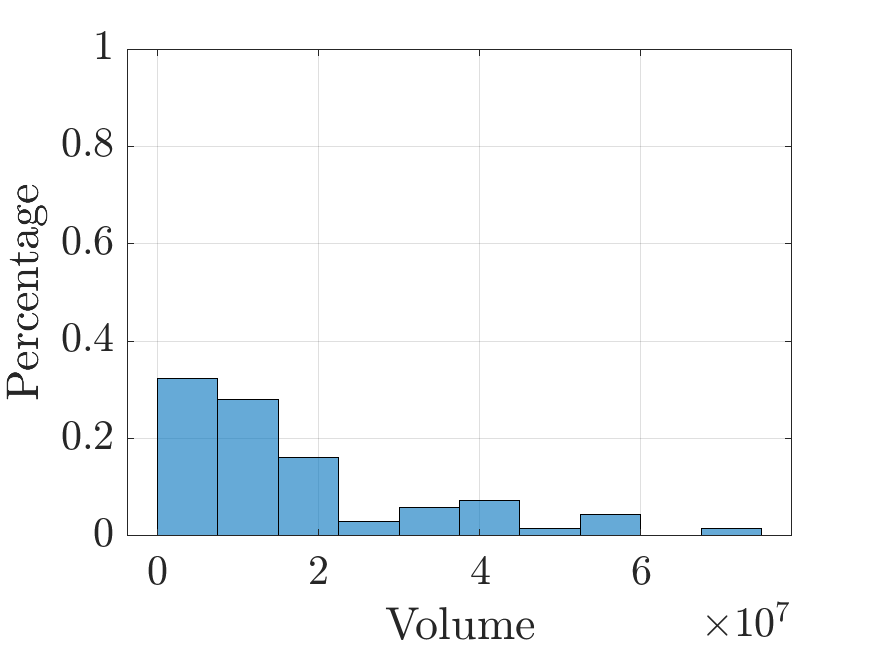**(g-2)** | |
| 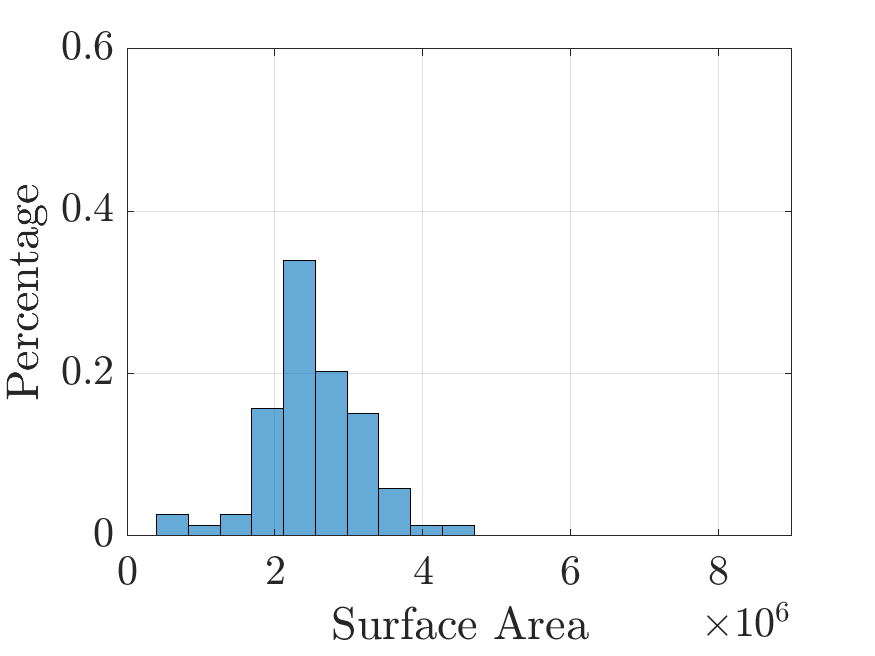**(h-1)** | | 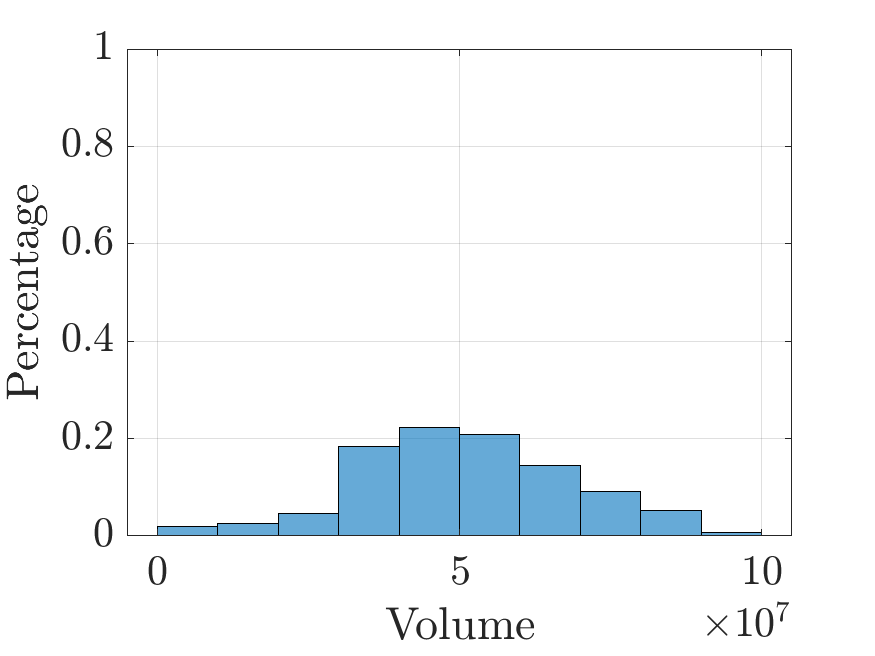**(h-2)** | |
| **(i-1)**  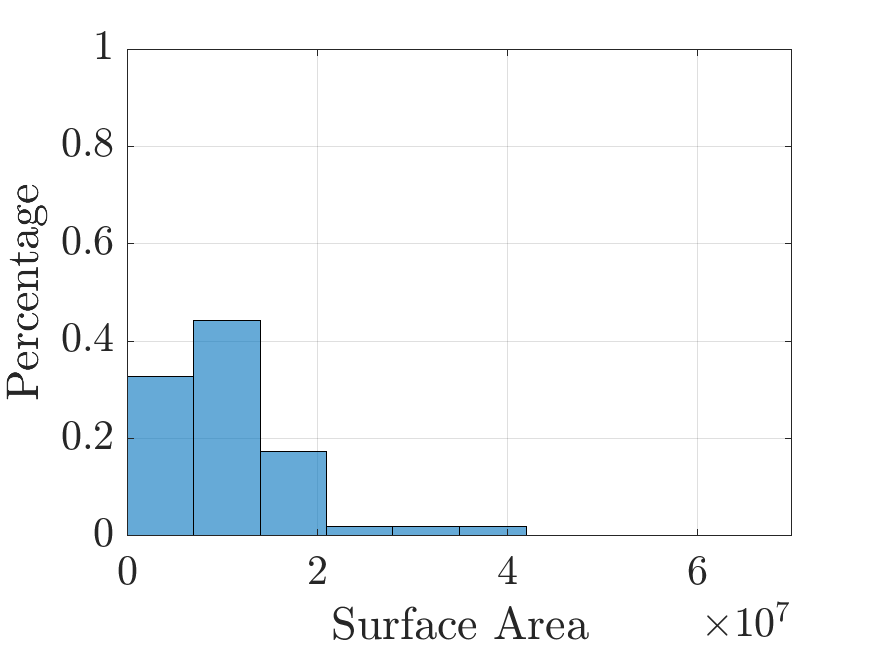 | | 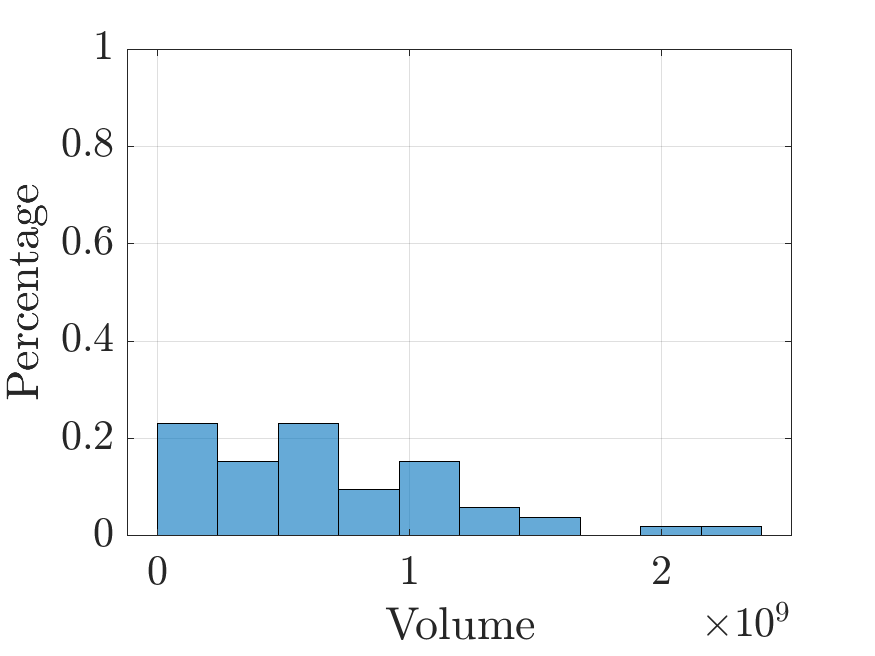**(i-2)** | |
| 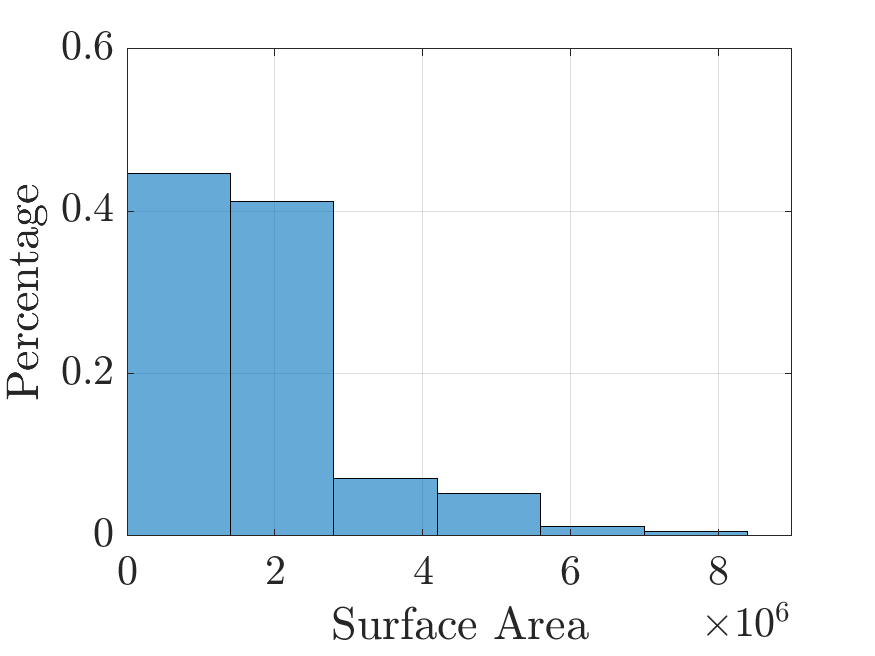**(j-1)** | | 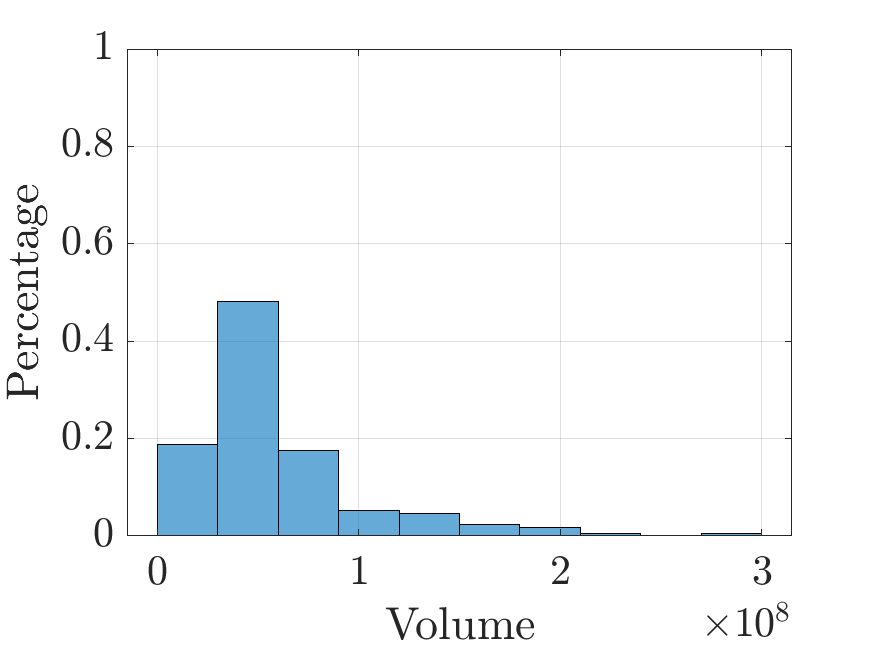**(j-2)** | |
| *Particle shape characterisation for* ***(a)****British rail sand,* ***(b)****Austrian rail sand,* ***(c)****waste glass beads,* ***(d)****dolomite,* ***(e)****Recycled crushed glass retained on 2 mm mesh sieve,* ***(f)****Recycled crushed glass retained on 1.18 mm mesh sieve,* ***(g)****Recycled crushed glass retained on 600 µm mesh sieve,* ***(h)****non-coated alumina,* ***(i)****coarse coated alumina, and* ***(j)****fine coated alumina based* ***(1)****surface area, and* ***(2)****volume obtained from X-ray Computed Tomography.* | | | |
| **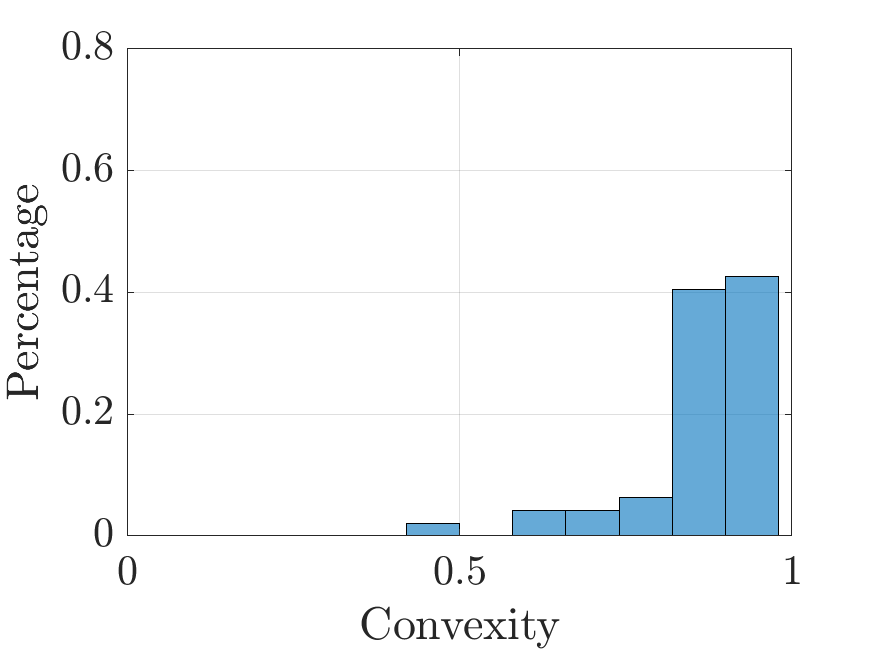(a-1)** | **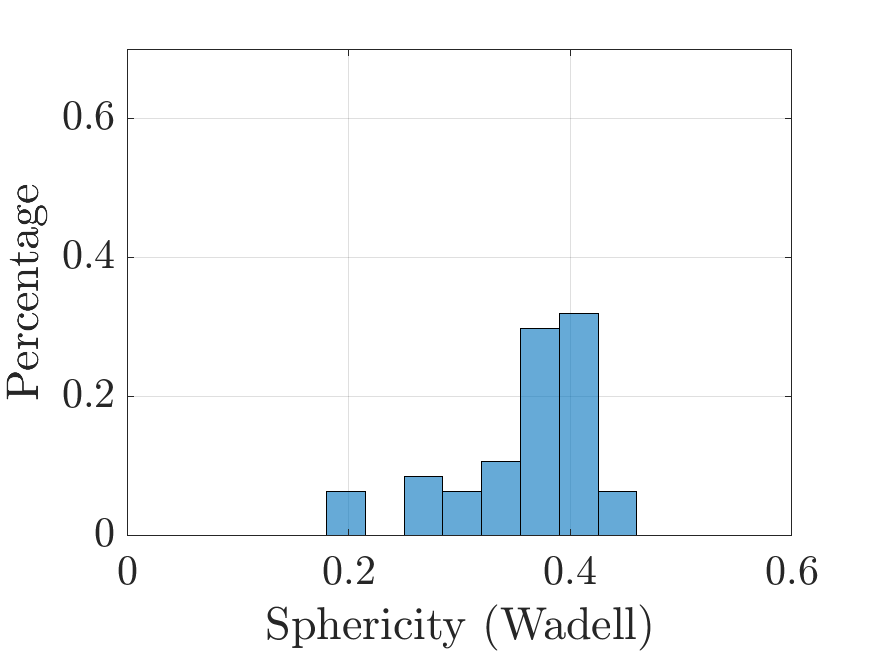(a-2)** | | **(a-3)**  **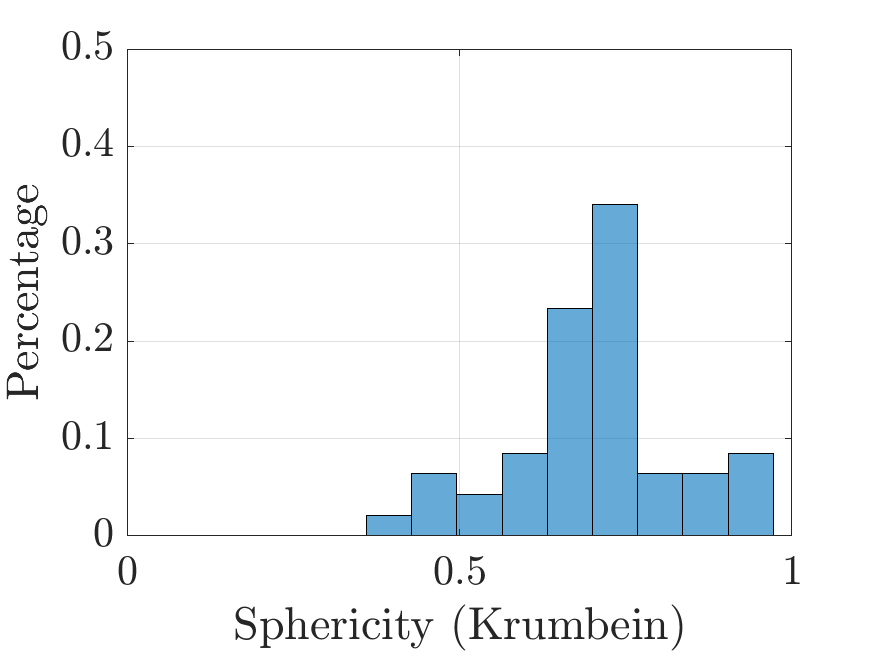** |
| 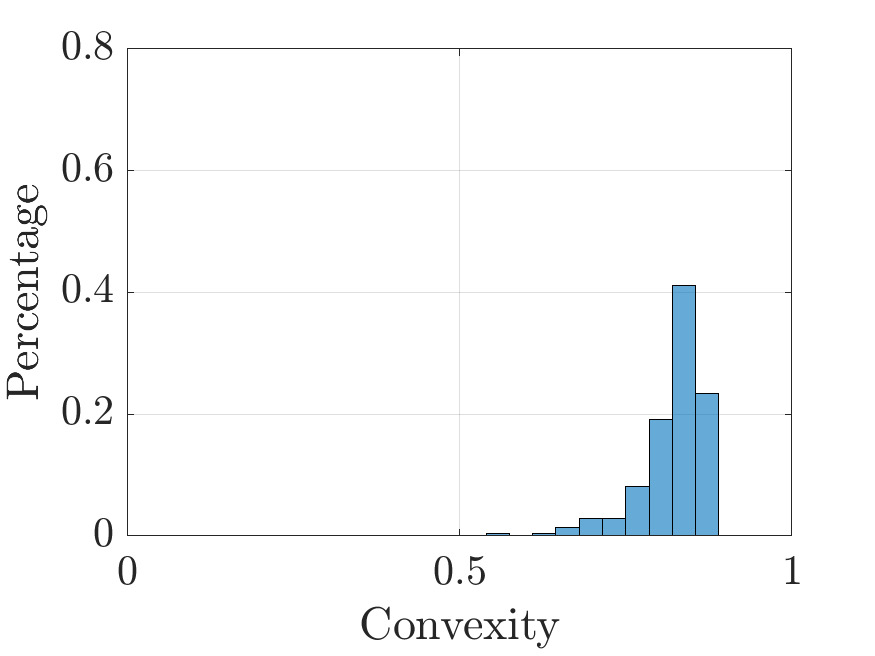**(b-1)** | **(b-2)**  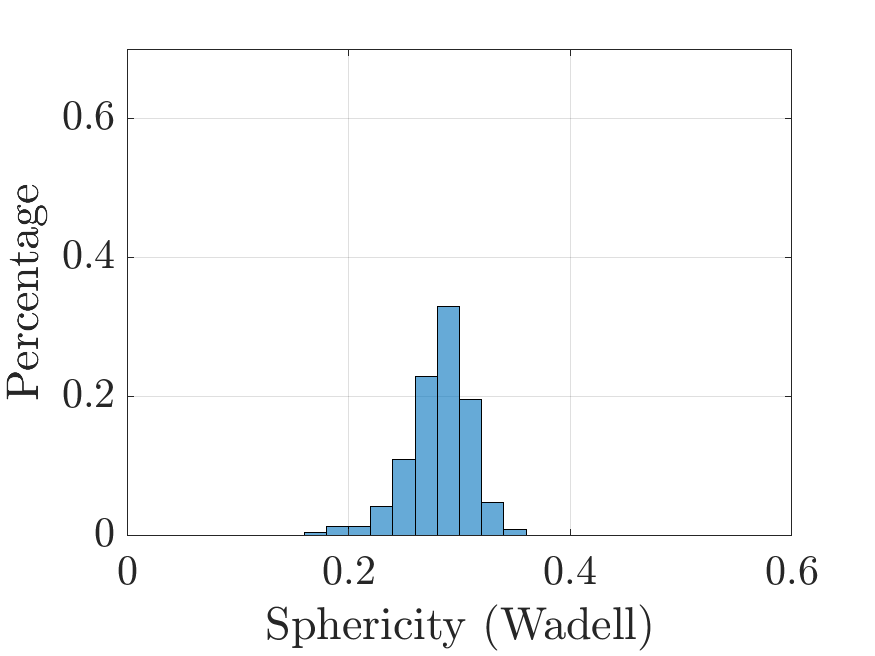 | | 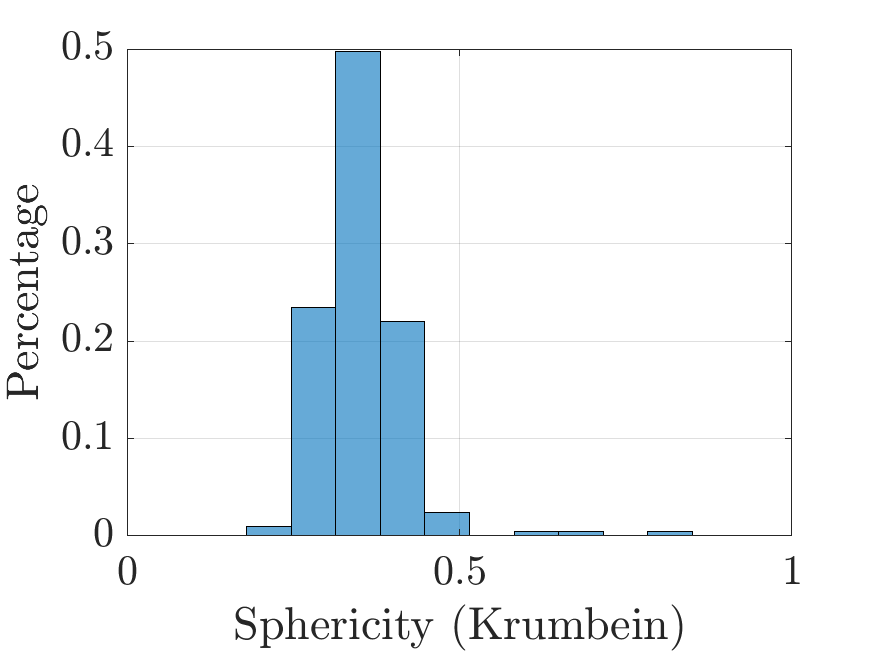**(b-3)** |
| 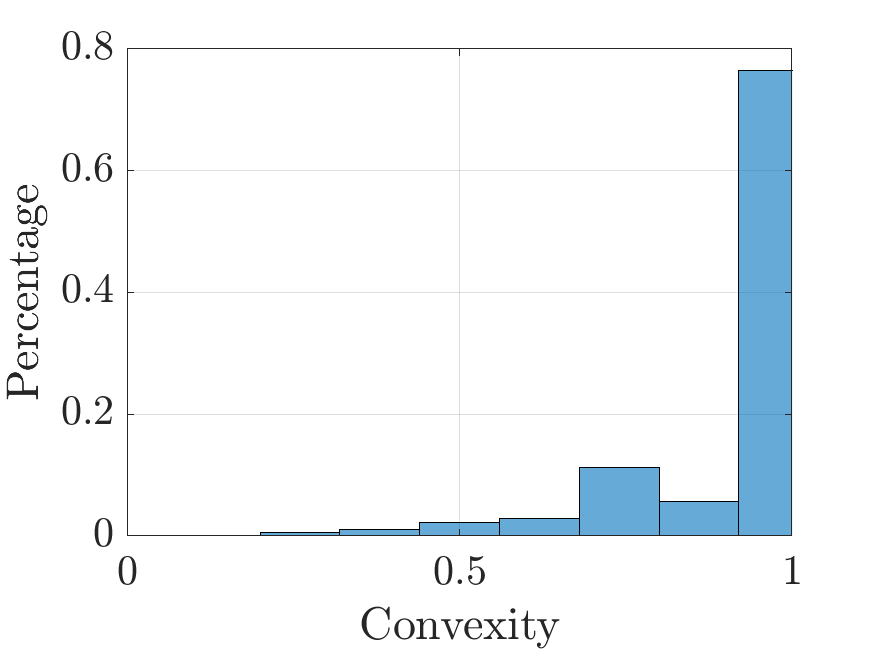**(c-1)** | 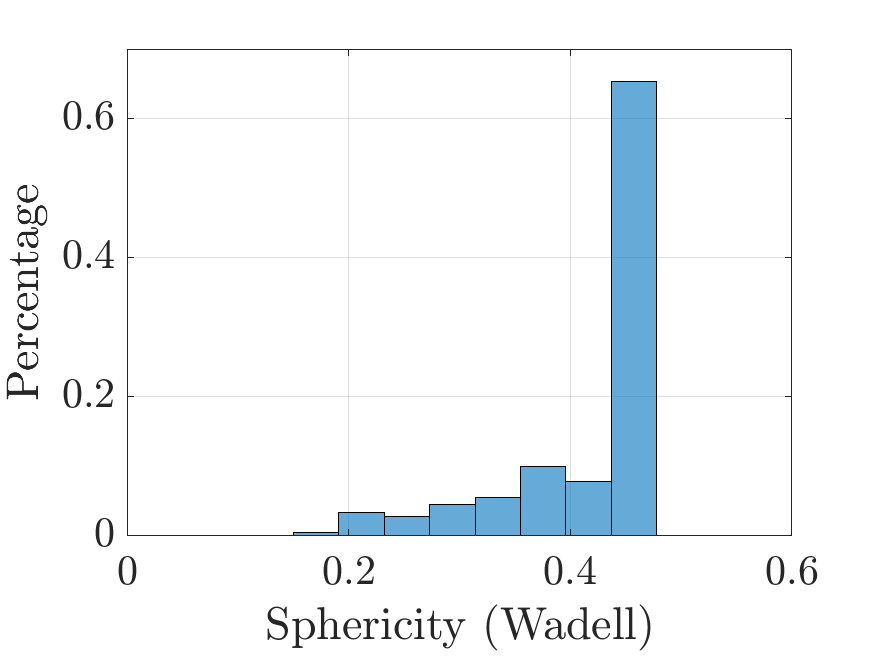**(c-2)** | | 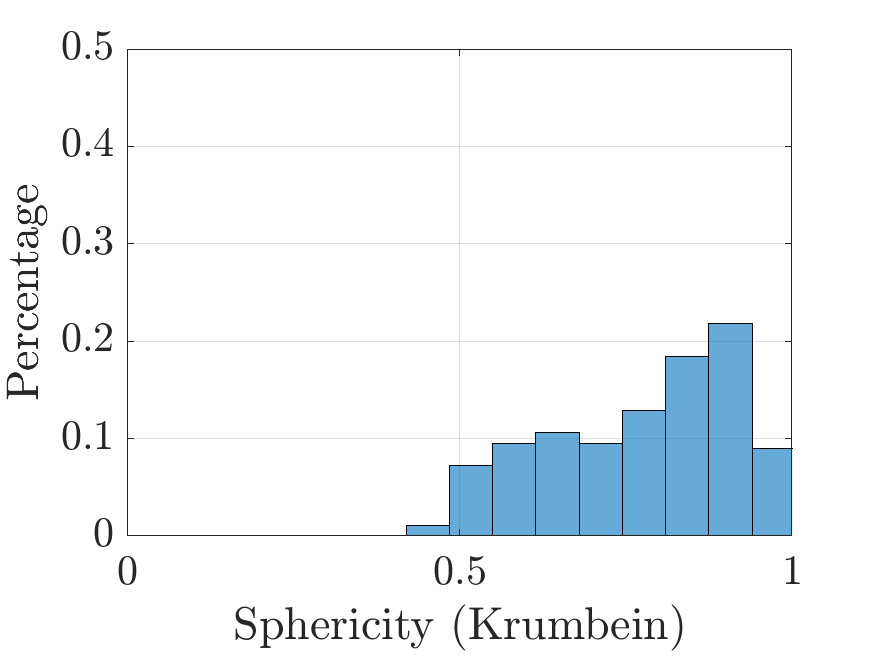**(c-3)** |
| 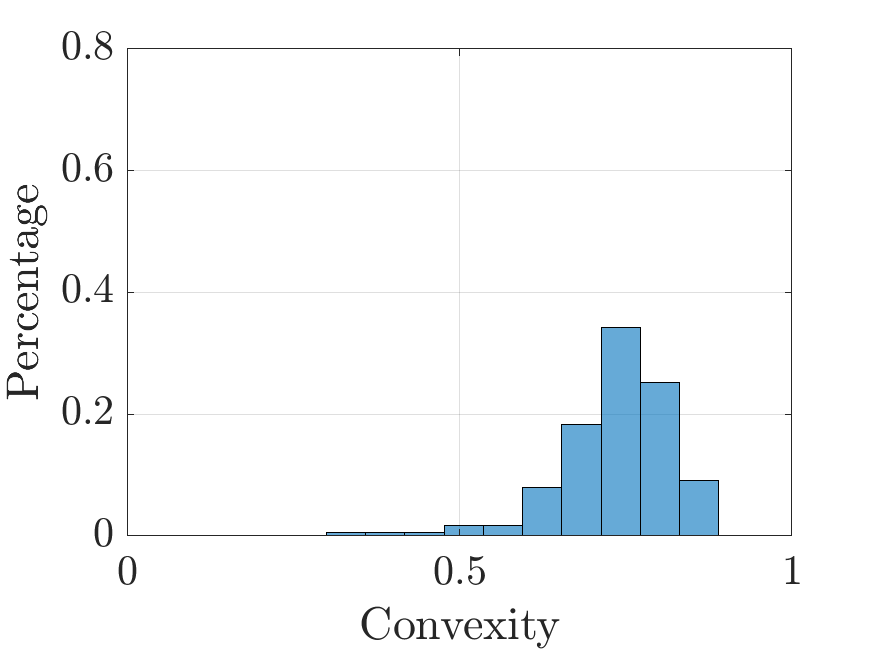**(d-1)** | 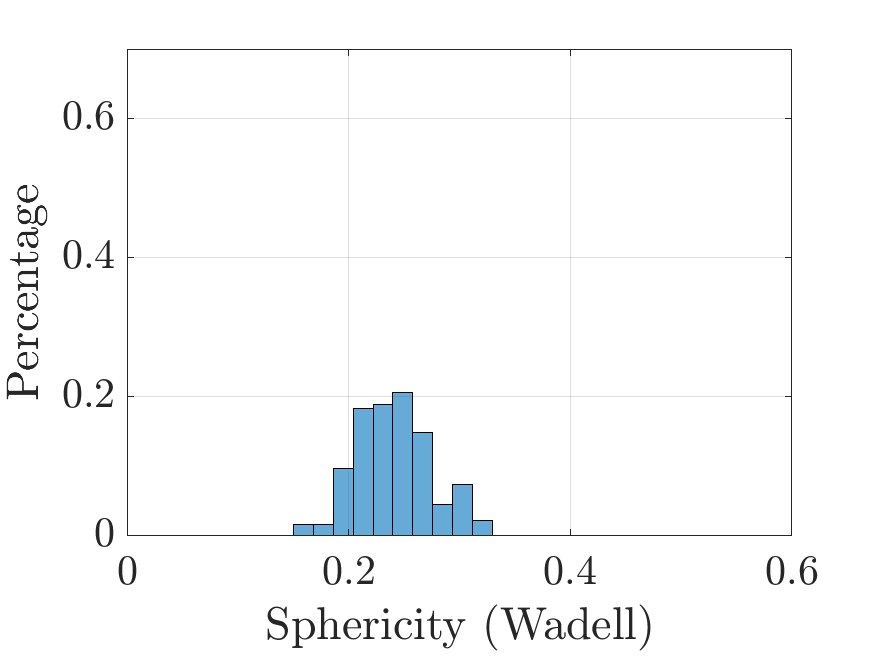**(d-2)** | | 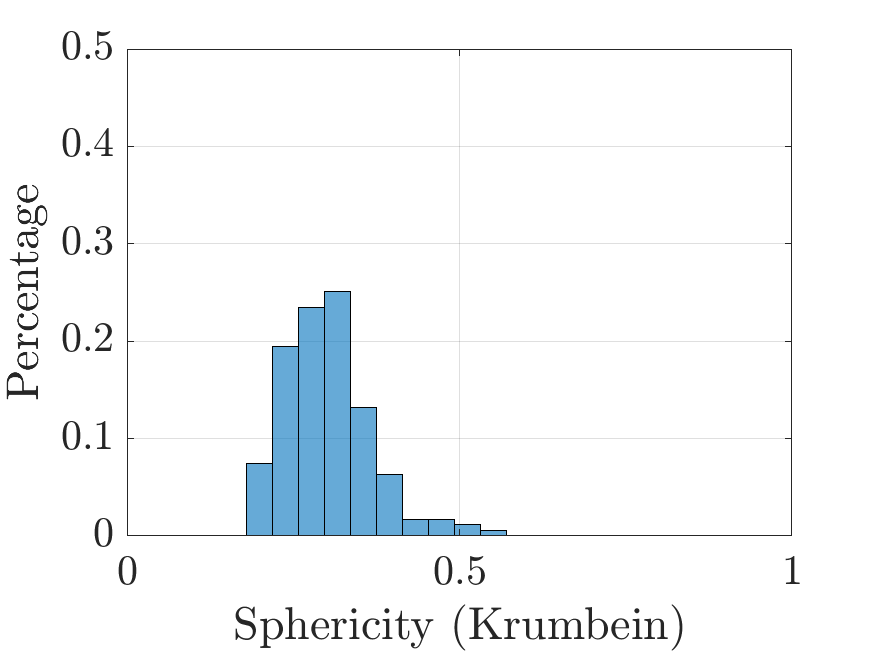**(d-3)** |
| **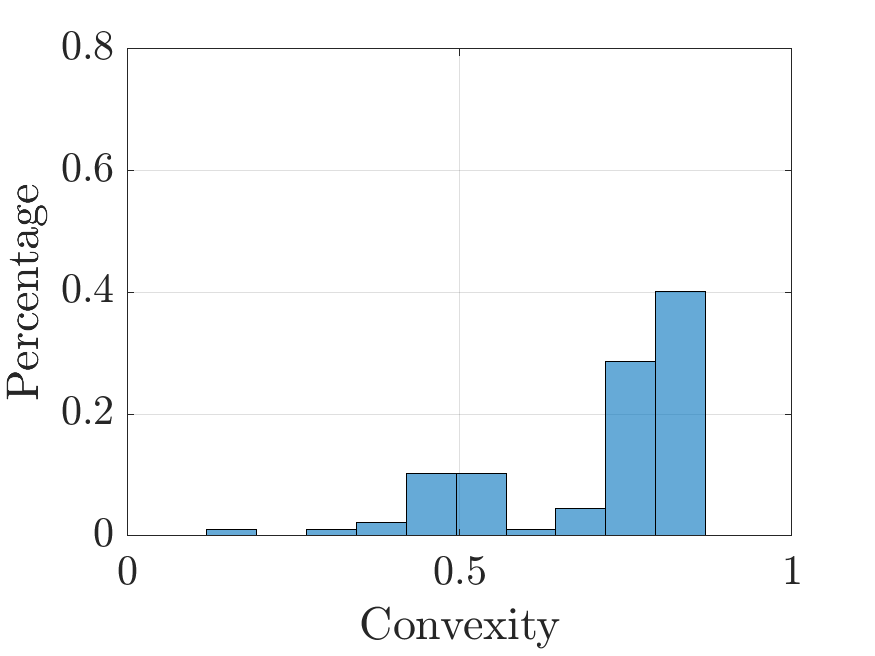(e-1)** | **(e-2)**  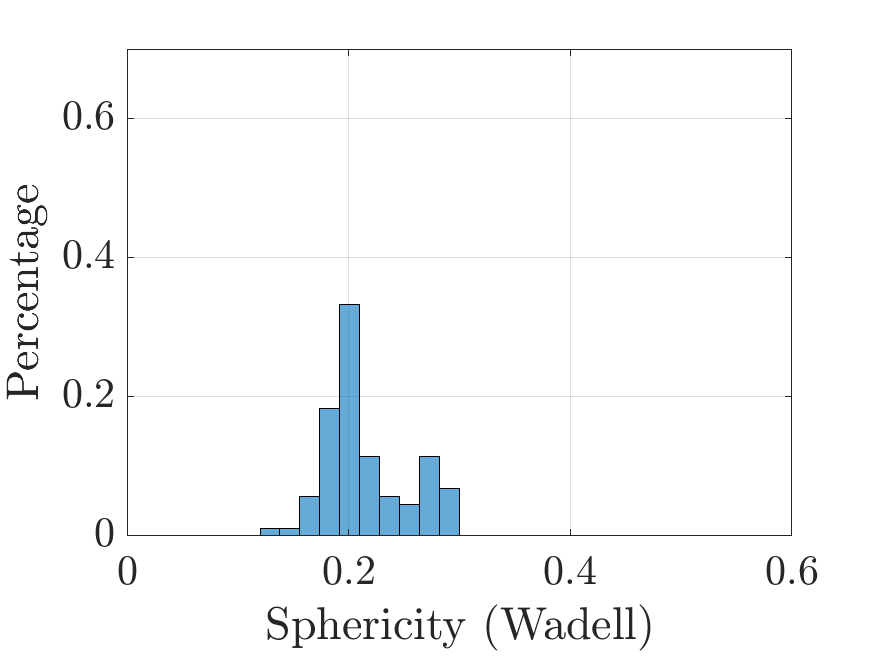 | | **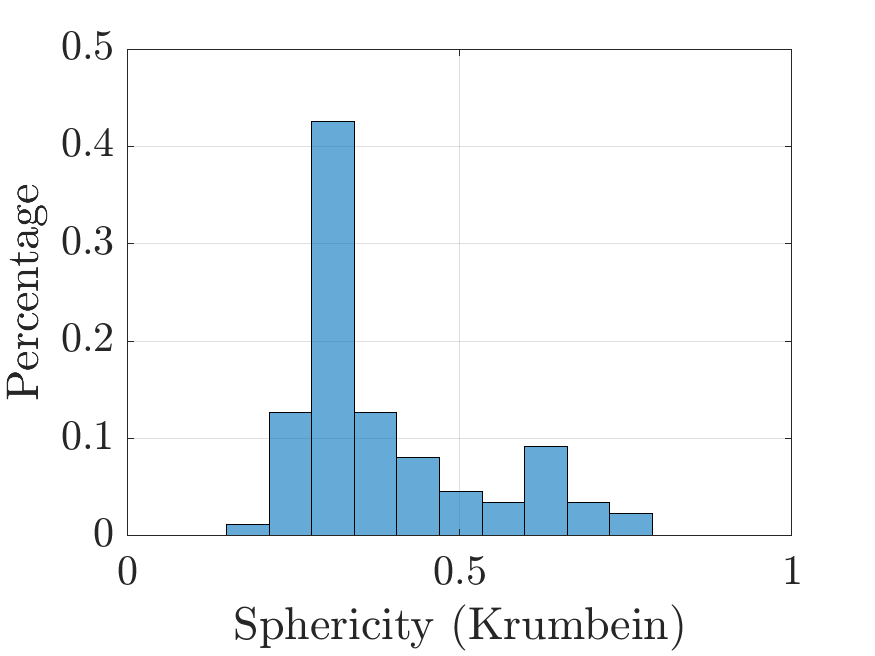(e-3)** |
| **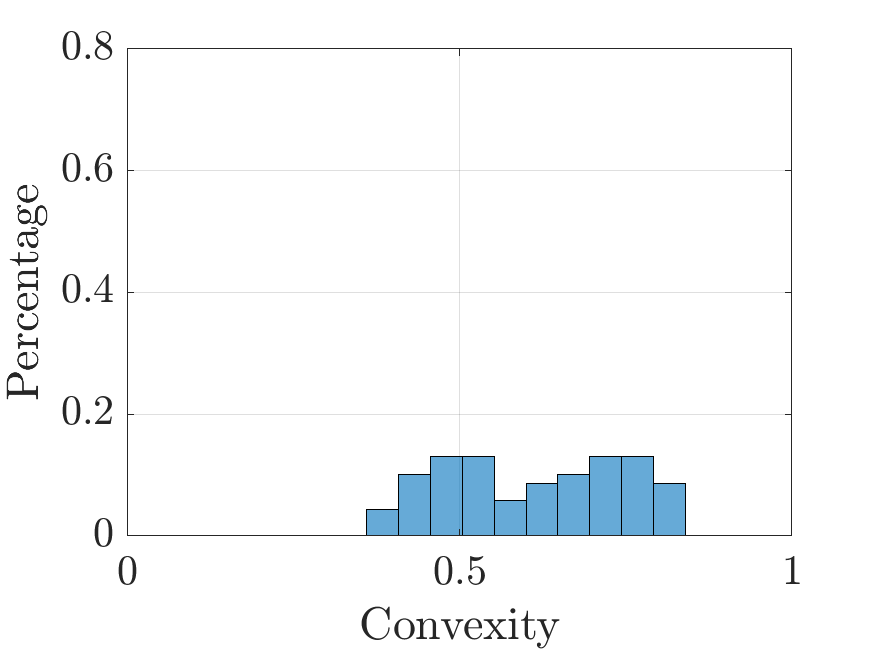(f-1)** | **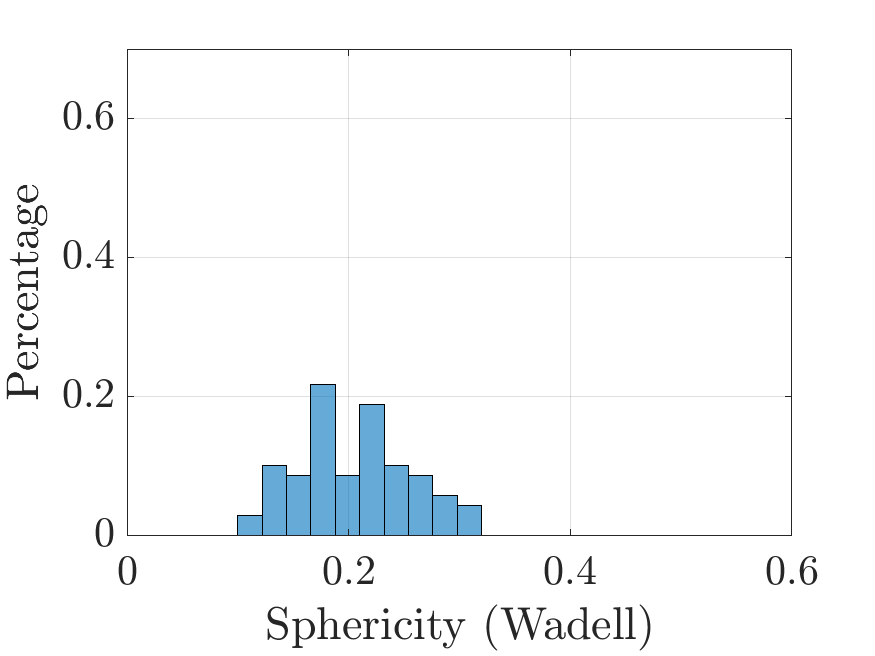(f-2)** | | **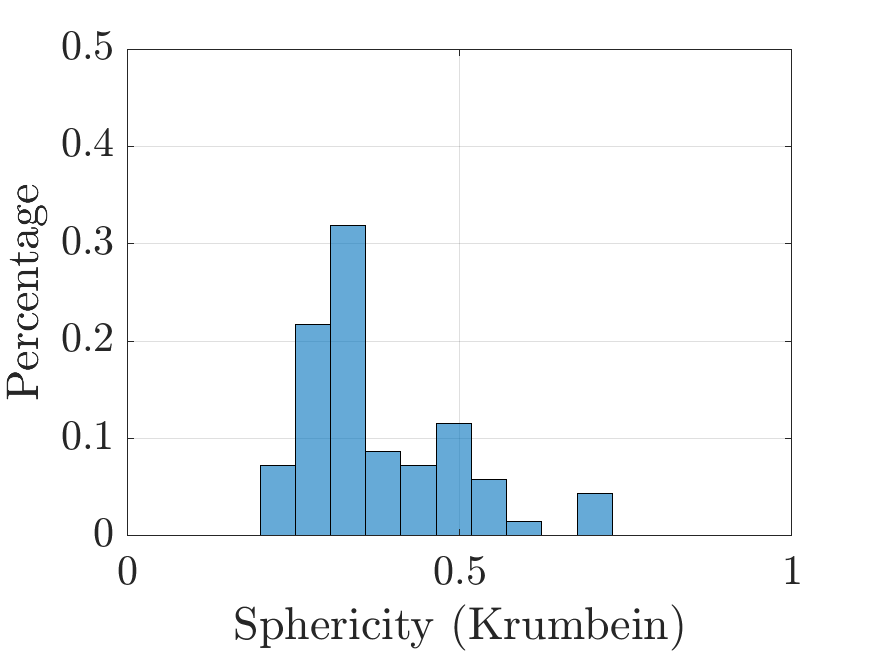(f-3)** |
| **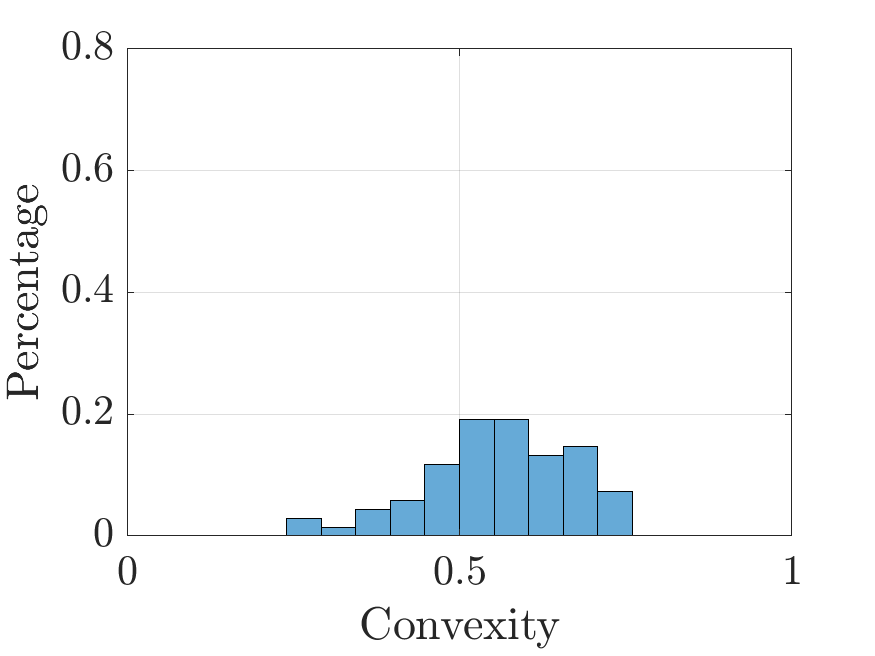(g-1)** | **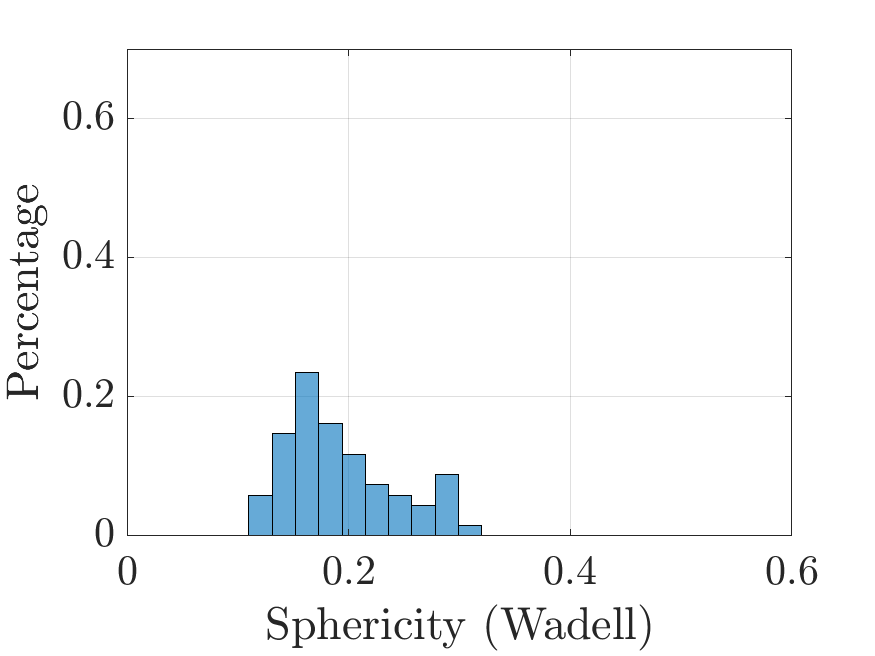(g-2)** | | **(g-3)**  **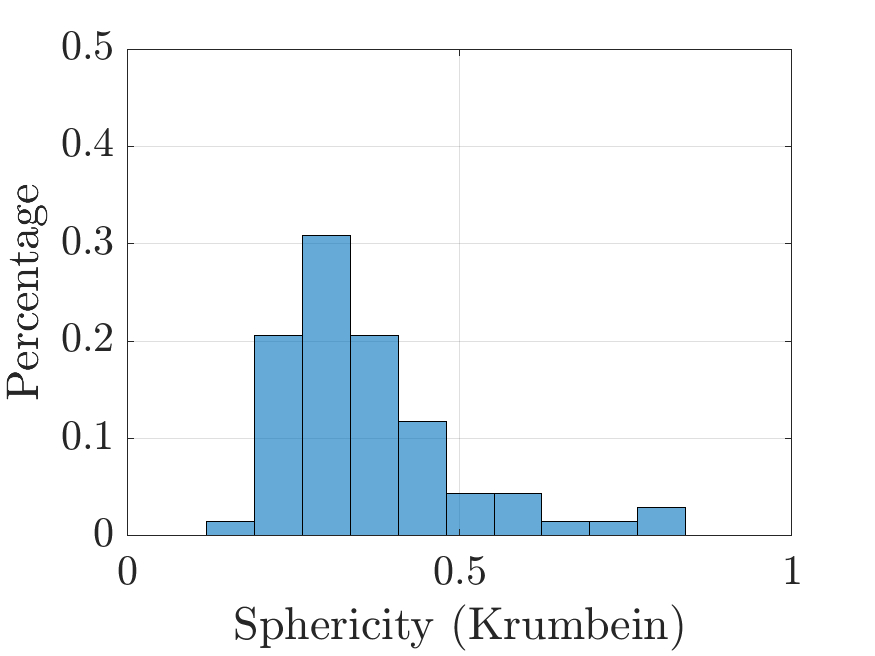** |
| **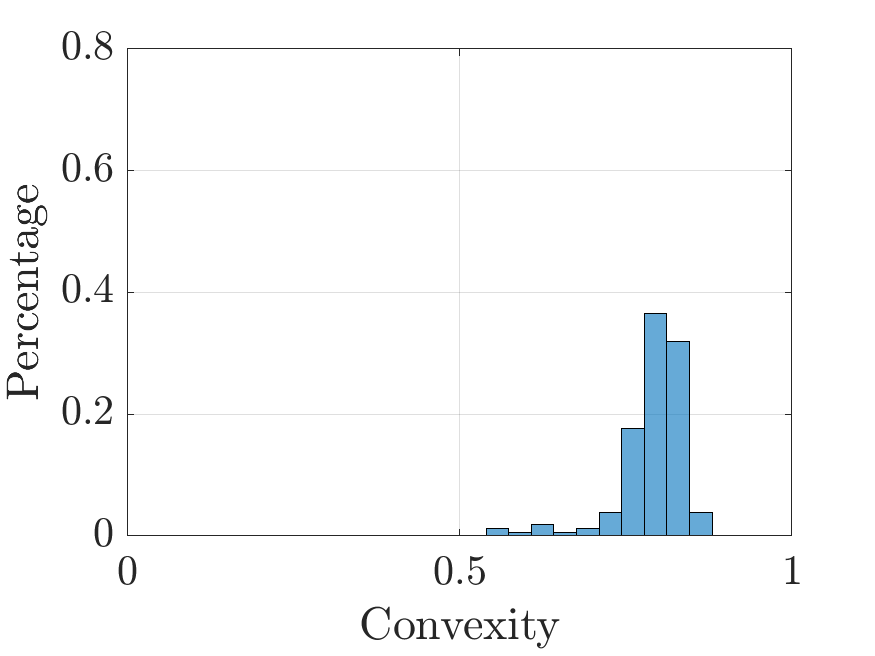(h-1)** | **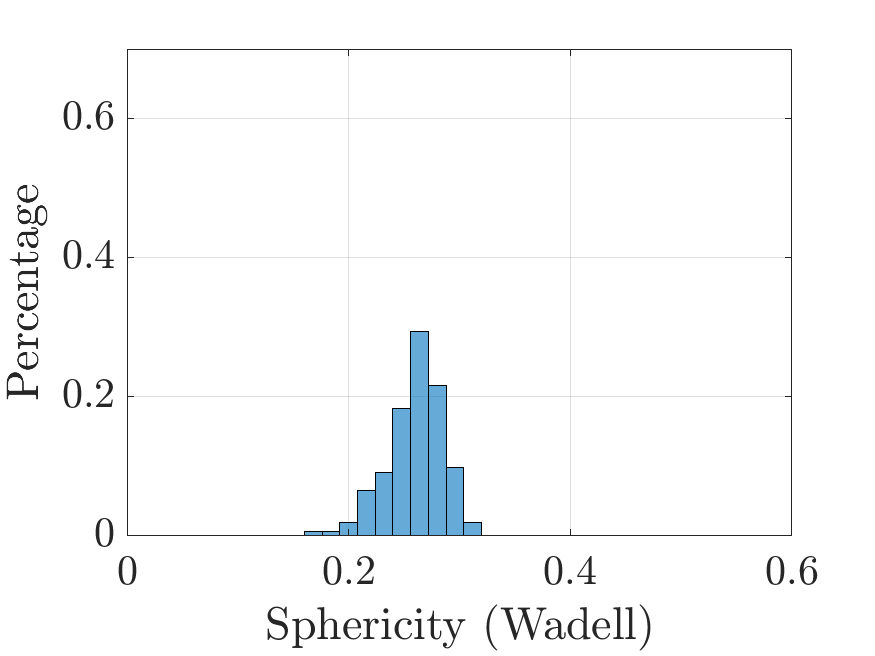(h-2)** | | **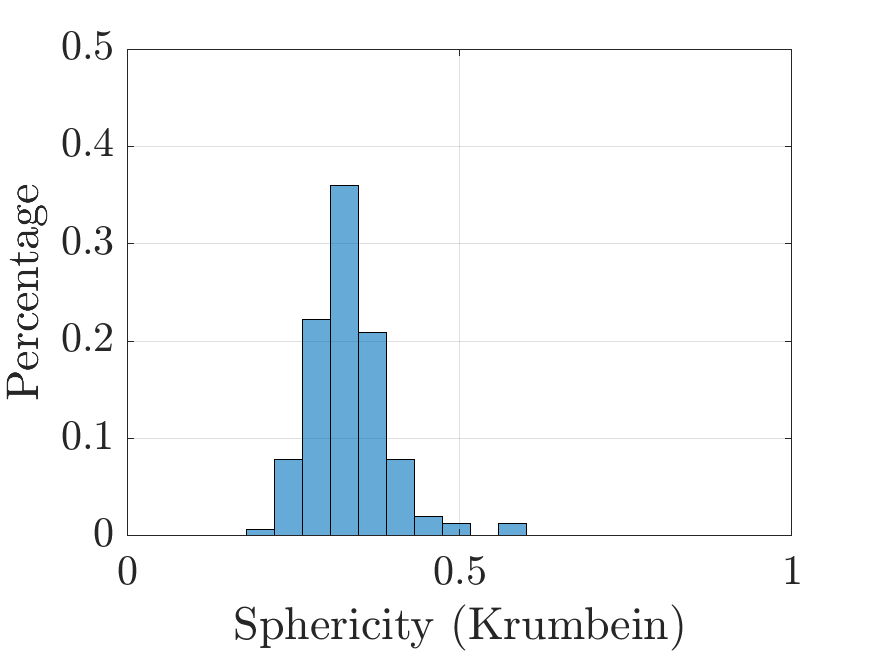(h-3)** |
| **(i-1)**  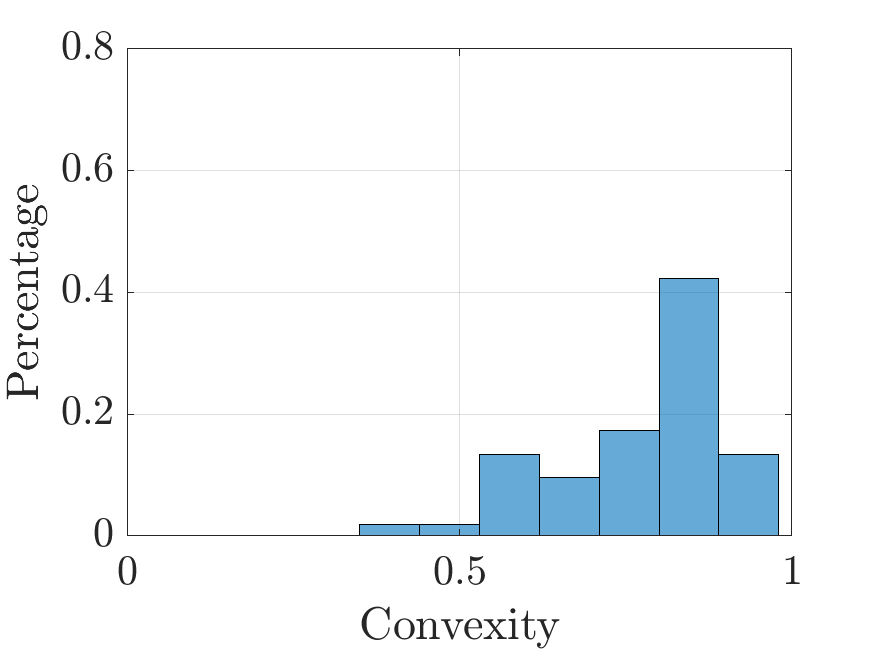 | **(i-2)**  **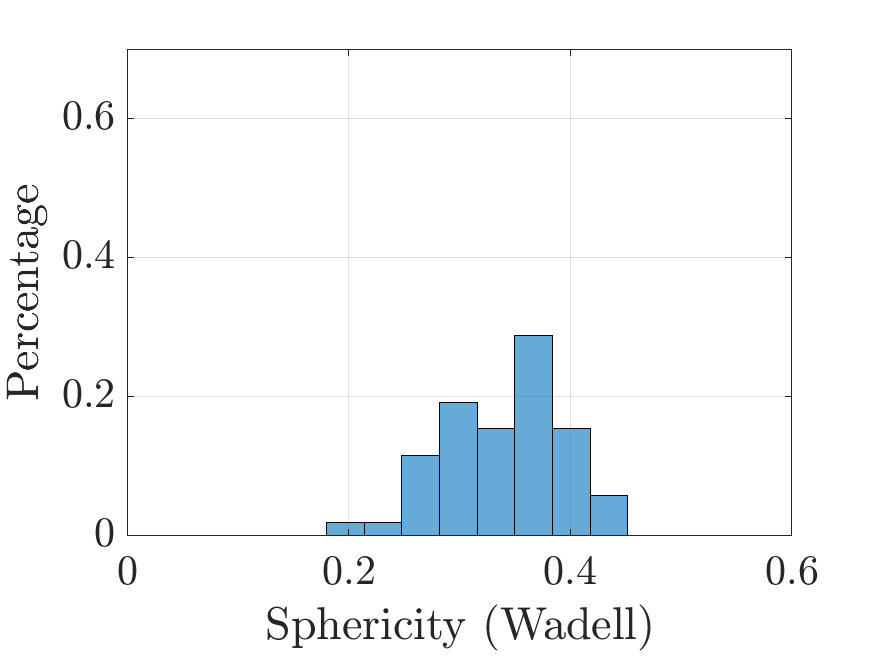** | | **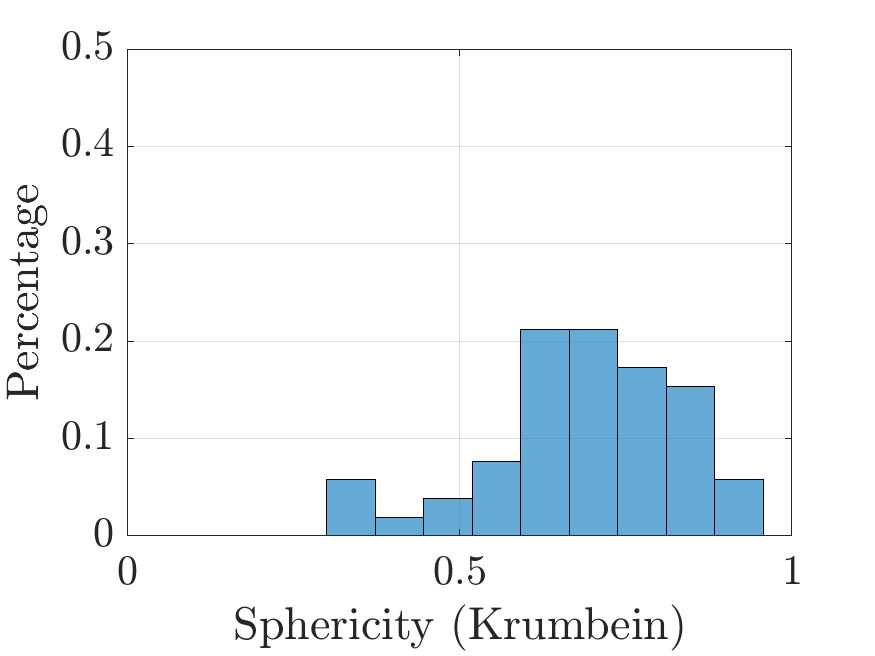(i-3)** |
| **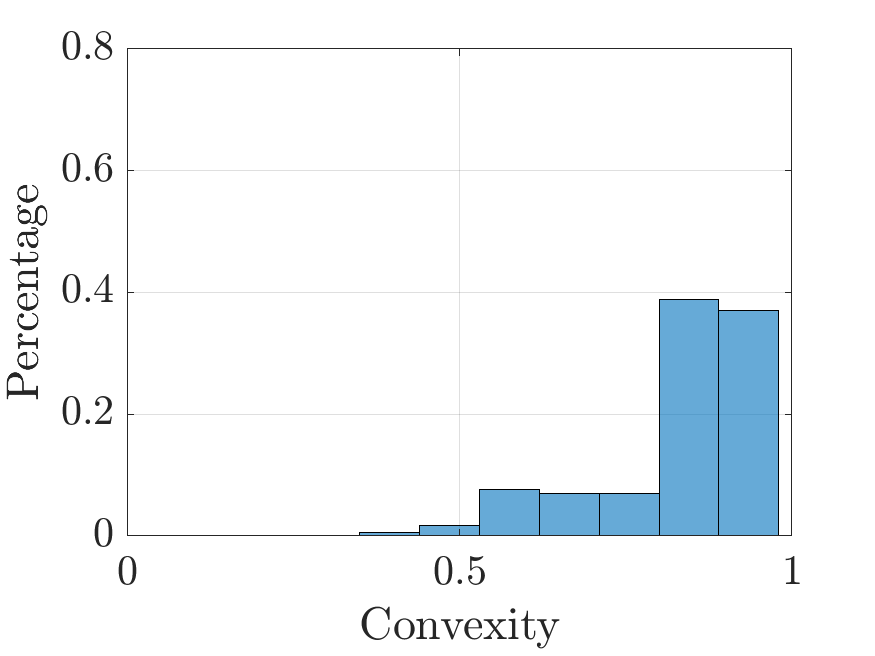(j-1)** | **(j-2)**  **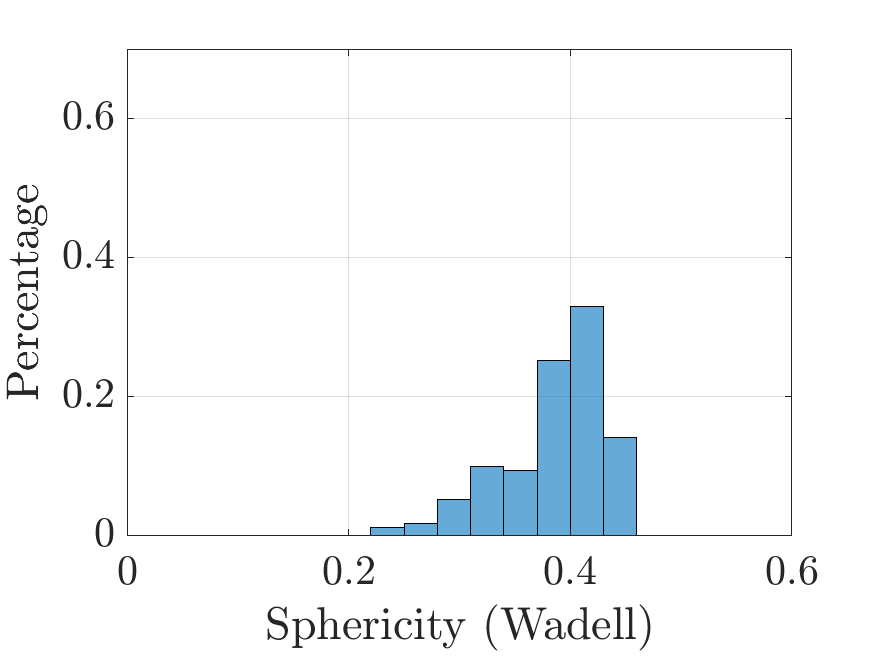** | | **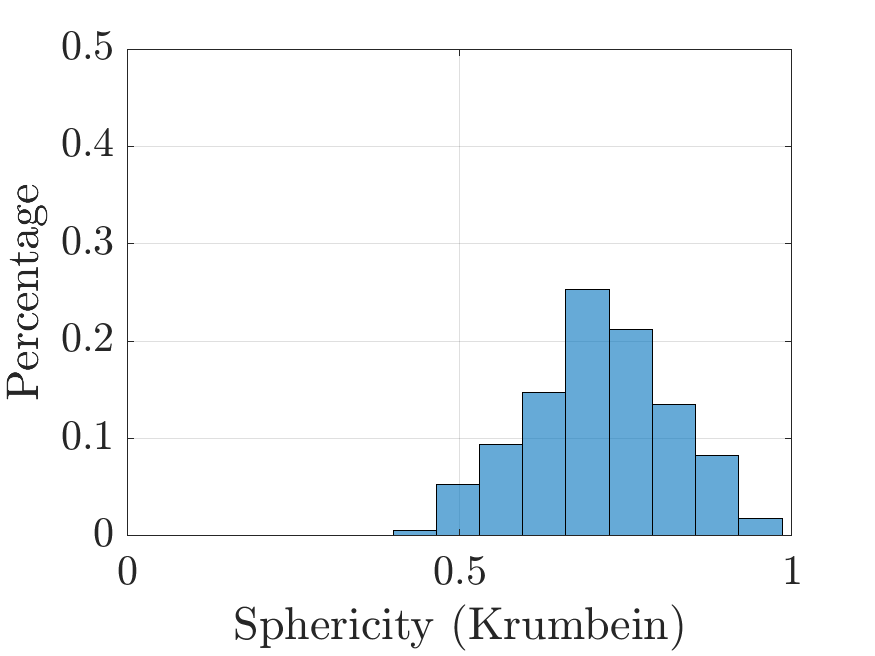(j-3)** |
| *Particle shape characterisation for* ***(a)****British rail sand,* ***(b)****Austrian rail sand,* ***(c)****waste glass beads,* ***(d)****dolomite,* ***(e)****Recycled crushed glass retained on 2 mm mesh sieve,* ***(f)****Recycled crushed glass retained on 1.18 mm mesh sieve,* ***(g)****Recycled crushed glass retained on 600 µm mesh sieve,* ***(h)****non-coated alumina,* ***(i)****coarse coated alumina, and* ***(j)****fine coated alumina based* ***(1)****convexity,* ***(2)****Wadell’s sphericity ^4^, and* ***(3)****Krumbein’s sphericity ^5^ and obtained from X-ray Computed Tomography.* | | | |

# References

1 Zingg, T. *Beitrag zur Schotteranalyse*, ETH Zurich, (1935).

2 Angelidakis, V., Nadimi, S. & Utili, S. SHape Analyser for Particle Engineering (SHAPE): Seamless Characterisation and Simplification of Particle Morphology from Imaging Data. *Computer Physics Communications* **265**, 107983 (2021). <https://doi.org:10.1016/j.cpc.2021.107983>

3 Angelidakis, V., Nadimi, S. & Utili, S. Elongation, Flatness and Compactness Indices to Characterise Particle Form. *Powder Technology* **396**, 689-695 (2022).

4 Wadell, H. Volume, Shape, and Roundness of Rock Particles. *The Journal of Geology* **40**, 443-451 (1932).

5 Krumbein, W. C. Measurement and Geological Significance of Shape and Roundness of Sedimentary Particles. *Journal of Sedimentary Research* **11**, 64-72 (1941).
